# Low blood levels of selenium, selenoprotein P and GPx3 are associated with accelerated biological aging: results from the Berlin Aging Study II (BASE-II)

**DOI:** 10.1186/s13148-025-01863-7

**Published:** 2025-04-25

**Authors:** Valentin Max Vetter, Kamil Demircan, Jan Homann, Thilo Samson Chillon, Michael Mülleder, Orr Shomroni, Elisabeth Steinhagen-Thiessen, Markus Ralser, Christina M. Lill, Lars Bertram, Lutz Schomburg, Ilja Demuth

**Affiliations:** 1 Department of Endocrinology and Metabolic Diseases (Including Division of Lipid Metabolism), Lipid Clinic at the Interdisciplinary Metabolism Center, Biology of Aging Working Group, Charité – Universitätsmedizin Berlin, Corporate Member of Freie Universität Berlin and Humboldt-Universität zu Berlin, Augustenburger Platz 1, 13353 Berlin, Germany; 2 Max Rubner Center (MRC) for Cardiovascular Metabolic Renal Research, Institute for Experimental Endocrinology, Charité - Universitätsmedizin Berlin, Corporate Member of Freie Universität Berlin and Humboldt-Universität zu Berlin, 10115 Berlin, Germany; 3 Institute of Epidemiology and Social Medicine, University of Münster, Münster, Germany; 4 Ageing Epidemiology Research Unit (AGE), School of Public Health, Imperial College London, London, UK; 5 Core Facility High Throughput Mass Spectrometry, Charité - Universitätsmedizin Berlin, Corporate Member of Freie Universität Berlin and Humboldt-Universität zu Berlin, Berlin, Germany; 6 Nuffield Department of Medicine, The Centre for Human Genetics, University of Oxford, Oxford, UK; 7 Lübeck Interdisciplinary Platform for Genome Analytics (LIGA), University of Lübeck, Lübeck, Germany; 8 Berlin Institute of Health at Charité – Universitätsmedizin Berlin, BCRT – Berlin Institute of Health Center for Regenerative Therapies, Berlin, Germany

## Abstract

**Background:**

Biological age reflects inter-individual differences in biological function and capacity beyond chronological age. DNA methylation age (DNAmA) and its deviation from chronological age, DNAmA acceleration (DNAmAA), which was calculated as residuals of leukocyte cell count adjusted linear regression of DNAmA on chronological age, were used to estimate biological age in this study. Low levels of serum selenium, selenoprotein P (SELENOP), and the selenocysteine-containing glutathione peroxidase 3 (GPx3) are associated with adverse health outcomes and selenium supplementation is discussed as an anti-aging intervention.

**Methods:**

In this study, we cross-sectionally analyzed 1568 older participants from the observational Berlin Aging Study II (mean age ± SD: 68.8 ± 3.7 years, 51% women). Serum selenium was measured by total reflection X-ray fluorescence (TXRF) spectroscopy and SELENOP was determined by sandwich ELISA. GPx3 was assessed as part of a proteomics dataset using liquid chromatography–mass spectrometry (LC–MS). The relationship between selenium biomarkers and epigenetic clock measures was analyzed using linear regression analyses. *P* values and 95% confidence intervals (not adjusted for multiple testing) are stated for each analysis.

**Results:**

Participants with deficient serum selenium levels (< 90 μg/L) had a higher rate of biological aging (DunedinPACE, *β* = − 0.02, SE = 0.01, 95% CI − 0.033 to − 0.004, *p* = 0.010, *n* = 865). This association remained statistically significant after adjustment for age, sex, BMI, smoking, and first four genetic principal components (*β* = − 0.02, SE = 0.01, 95% CI − 0.034 to − 0.004, *p* = 0.012, *n* = 757). Compared to the highest quartile, participants in the lowest quartile of SELENOP levels showed an accelerated biological aging rate (DunedinPACE, *β* = − 0.03, SE = 0.01, 95% CI − 0.051 to − 0.008, *p* = 0.007, *n* = 740, fully adjusted model). Similarly, after adjustment for confounders, accelerated biological age was found in participants within the lowest GPx3 quartile compared to participants in the fourth quartile (DunedinPACE, *β* = − 0.04, SE = 0.01, 95% CI − 0.06 to − 0.02, *p* = 0.001, *n* = 674 and GrimAge, *β* = − 0.98, SE = 0.32, 95% CI − 1.6 to − 0.4, *p* = 0.002, *n* = 608). Only the association with GPx3 remained statistically significant after multiple testing correction.

**Conclusion:**

Our study suggests that low levels of selenium biomarkers are associated with accelerated biological aging measured through epigenetic clocks. This effect was not substantially changed after adjustment for known confounders.

**Supplementary Information:**

The online version contains supplementary material available at 10.1186/s13148-025-01863-7.

## Background

Over the past few decades the average lifespan increased faster than the healthspan, which resulted in a growing burden of late-life diseases [1, 2]. However, there are substantial inter-individual differences in the pace of aging and some people remain disease-free and in good health until old age. Although some lifestyle habits were shown to impact some areas of aging [3] they do not fully explain the observed between-person differences. Hence, the identification of additional factors promoting a “healthy aging” trajectory would potentially benefit many. One recent, and often considered very useful [4, 5] biomarker to quantify difference in biological aging is DNA methylation age (DNAmA, epigenetic age) estimated from DNA methylation data by so called epigenetic clocks [6]. The deviation of epigenetic age from chronological age, DNAmA acceleration (DNAmAA), was shown in previous studies to be associated with mortality as well as with age-related diseases and phenotypes [6]. First-generation clocks (e.g., Horvath DNAmA [7], Hannum DNAmA [8], 7-CpG DNAmA [9]) were trained to predict chronological age. The difference between actual and predicted chronological age, DNAmA acceleration (DNAmAA), was shown to be associated with mortality as well as with numerous age-related diseases and phenotypes [6]. To further improve the predictive ability of the epigenetic clock algorithms, the second generation of clocks was trained to predict composite markers of different meaningful age-associated variables including serum proteins, smoking and others (e.g., GrimAge [10], PhenoAge [11]). One of the latest epigenetic clocks, the DundinPACE clock [12], was developed to capture the change in biomarkers and health indicators over a span of 19-years. In some studies, this third-generation clock was shown to pick up biological information better than the previously developed clocks [12–14]. One of the main advantages of using epigenetic clocks is, that a general biological state of an individual can be assessed which is associated with health outcomes that would become measurable only much later in life. Therefore, time and costs are reduced as now much shorter follow-up periods can produce valuable estimations of long-term effects. Due to the ability of these clocks to assess the biological age of an individual they are increasingly used as endpoints for the evaluation of anti-aging interventions (reviewed in [14–18]).

In related research, one factor discussed in the context of healthy aging is selenium, an essential trace element that regulates thyroid hormone metabolism, antioxidative, and redox processes through incorporation into selenoproteins [19], which needs to be present in sufficient levels to exert its physiological function. Selenium status and its cellular availability (determined by dietary intake and its absorption) directly regulate expression and activity of selenoproteins [20]. Selenium status can be determined by assessment of serum selenium biomarkers such as total selenium, the selenium transporter selenoprotein P (SELENOP), or glutathione peroxidase 3 (GPx3), a selenocysteine-containing extracellular antioxidant enzyme [21]. Emerging clinical evidence indicates an association between a deficient status of these selenium biomarkers and outcomes of mortality and chronic diseases of older age, i.e., measures of an increased pace of aging [22–26]. The direct association between selenium biomarkers and epigenetic aging, however, remains understudied. The relationship between blood selenium levels and DNAmA was analyzed in two previous studies, which revealed promising links between selenium levels and biological aging [27, 28]. However, both studies were of small sample size (*n* = 93 [27] and *n* = 276 [28]), and analyzed only total selenium, but none of the other selenium-related variables. In this study, we analyzed the association between biological aging as determined with three DNAmA estimators (i.e., Horvath [7], GrimAge [10], and DunedinPACE [12]) with selenium status determined by total serum selenium, and two complementary protein biomarkers, SELENOP, and GPx3, in a large cohort of 1568 older participants from the Berlin Aging Study II (BASE-II).

## Methods

### Study population

The Berlin Aging Study II (BASE-II) is a multi-disciplinary study that aims at the identification of factors promoting a healthy aging trajectory. The older sample of the medical BASE-II part consists of 1671 participants (60–85 years) which was recruited in the metropolitan area of Berlin, Germany, through advertisements in local newspapers and on public transport. Additionally, 500 younger participants between 20 and 37 years of age were assessed but are not analyzed here. Participants who reported the intake of selenium supplements were excluded (*n* = 103). As described below, DNA methylation data were measured with two different methods resulting in slightly higher sample sizes for analyses including the 7-CpG clock. A detailed flow chart on how we arrived at the final sample sizes is shown in Supplementary Fig. 1. Please refer to the study’s cohort profile for a detailed description of study design and data collection [29].

### DNA methylation age (DNAmA), DNAmA acceleration (DNAmAA) and pace of aging

The participants’ epigenetic age was estimated from DNA methylation data utilizing three “first-generation” clocks (Horvath [7], Hannum [8], 7-CpG [9]) and two “second-generation” clocks (PhenoAge [11], GrimAge [10]). The pace of aging representing the number of years of biological age passed for each year of chronological age was estimated using DunedinPACE [12], a third-generation clock. DNA was isolated from EDTA whole blood samples using the LGC “Plus XL manual kit” (LGC) and stored at − 80 °C. DNAm data for the 7-CpG clock were measured by Single Nucleotide Prime Extension (SNuPE) following a bisulfite conversion performed with “EZ-96 DNA Methylation-Lightning Kit” (ZYMO RESEARCH) in 1471 samples. A detailed laboratory protocol was published before [9]. All other clocks were calculated from *n* = 1,030 genome-wide DNA methylation profiles measured in the same DNA samples used for the SNuPE methods by the “Infinium MethylationEPIC” array (Illumina, Inc., USA). Methylation data underwent quality control and the raw methylation values were then uploaded to Steve Horvaths website to derive the DNAmA based on the Horvath, Hannum, PhenoAge, and GrimAge algorithm. The DunedinPACE clock was calculated based using the methods described in the original publication [12]. Additional information on laboratory procedures and data processing of the Illumina methylation data can be found in reference [30].

**Fig. 1:**
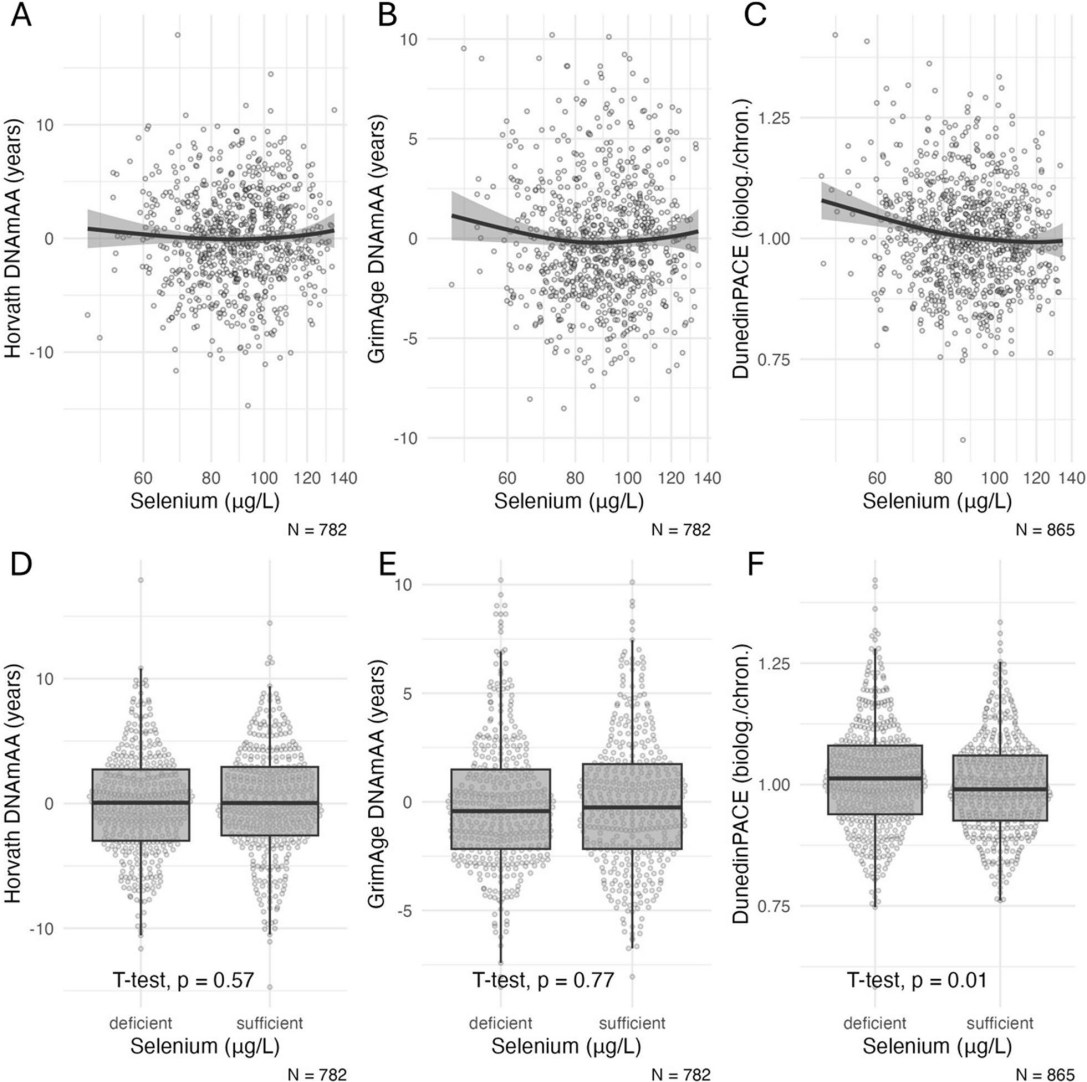
**A**–**C** Scatterplots of serum selenium levels at baseline and biological age estimators. The *x*-axis is log-scaled. All available participants of the older age group are included. **D**–**F** Boxplots of biological age estimators stratified by selenium status (deficient vs. sufficient. cut-off 90 μg/L) at baseline. Statistical significance of difference between group means was assessed by *t*-test. Note: DNAmAA = DNA methylation age acceleration; biolog. = biological years; chron. = chronological years. Boxplots show the median, hinges (corresponding to 25 th and 75 th percentile) and whiskers (1.5*inter-quartile range (IQR))

**Fig. 2:**
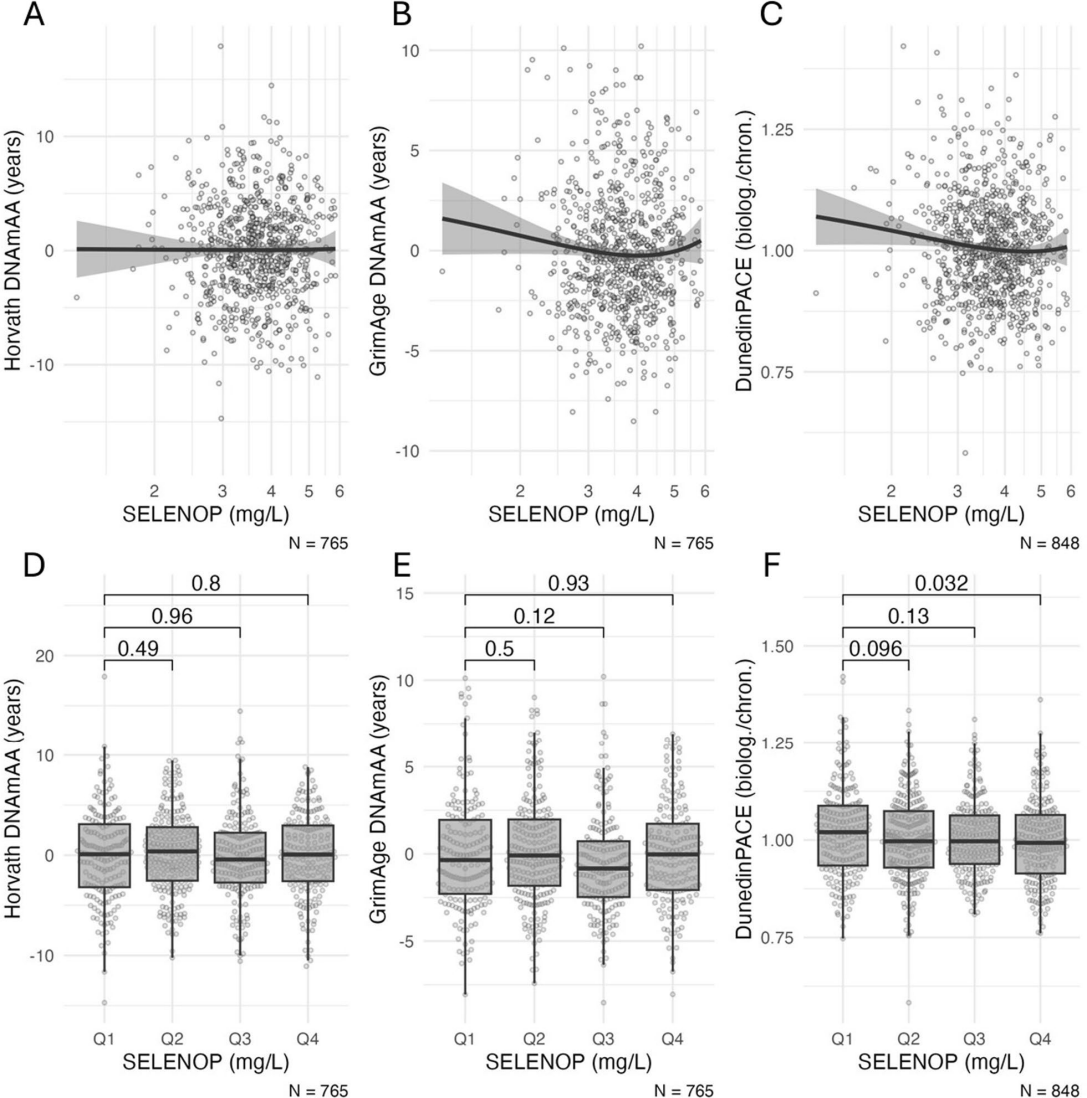
**A**–**C** Scatterplots of SELENOP levels and biological age estimators. The *x*-axis is log-scaled. **D**–**F** Boxplots of biological age estimators stratified by quartiles of SELENOP (Q1: 1.26–3.13 mg/L, Q2: 3.14–3.70 mg/L; Q3: 3.71–4.27, Q4: 4.28–5.86). Statistical significance of difference between group means was assessed by t-test. Note: DNAmAA = DNA methylation age acceleration; biolog. = biological years; chron. = chronological years. Boxplots show the median, hinges (corresponding to 25 th and 75 th percentile) and whiskers (1.5*inter-quartile range (IQR))

**Fig. 3:**
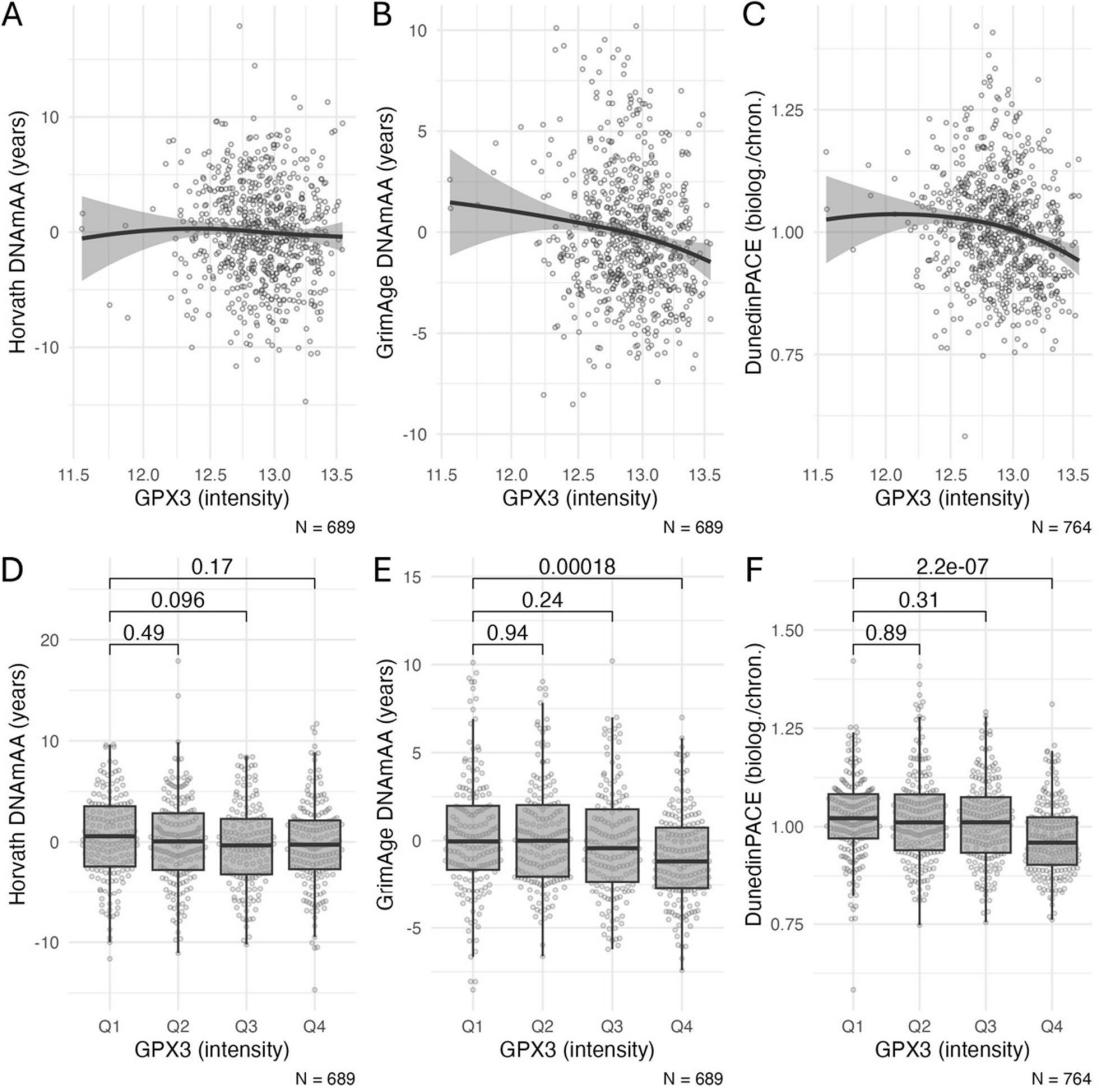
**A**–**C** Scatterplots of GPx3 intensity (proteomics data) and biological age estimators. The *x*-axis is log-scaled. **D**–**F** Boxplots of biological age estimators stratified by quartiles of GPx3 intensity (proteomics data, Q1: 11.6–12.7, Q2: 12.71–12.90, Q3: 12.91–13.10, Q3: 13.11–13.6). Statistical significance of difference between group means was assessed by t-test. Note: DNAmAA = DNA methylation age acceleration; biolog. = biological years; chron. = chronological years. Boxplots show the median, hinges (corresponding to 25 th and 75 th percentile) and whiskers (1.5*inter-quartile range (IQR))

DNA methylation age acceleration (DNAmAA) was calculated for all clocks (except for DunedinPACE) as residuals of a linear regression of DNAmA on chronological age accounting for leukocyte cell distribution (neutrophils, monocytes, lymphocytes, and eosinophils in G/l) (Table 1). Results for Horvath, GrimAge and DunedinPACE clock (Tables 2, 3 and 4, Figs. 1, 2, and 3) are shown as part of the main manuscript and analyses including 7-CpG, Hannum and PhenoAge clock are presented in the Supplementary Material.

**Table 1:** Baseline characteristics of analyzed sample

Variables	Mean (SD), *n* (%)	Min	Max	*n*
Age (years)	68.79 (3.73)	60.16	84.63	1568
Sex (women)	799 (51.0)			1568
BMI (kg/m^2^)	26.83 (4.19)	17.03	47.68	1534
Smoking (packyears)	10.51 (17.75)	0.00	148.00	1511
Serum Selenium (μg/L)	89.65 (17.38)	45.20	134.60	1357
Selenium deficiency (< 90 µg/L)	656 (48.3)			1357
SELENOP (mg/L)	3.72 (0.83)	1.26	5.86	1324
GPx3 (intensity)	12.52 (0.27)	11.33	13.11	1209
7-CpG DNAmAA (years)	0.00 (6.97)	− 22.93	26.61	1305
Horvath DNAmAA (years)	0.04 (4.27)	− 14.71	17.90	853
Hannum DNAmAA (years)	− 0.04 (3.48)	− 10.90	19.15	853
PhenoAge DNAmAA (years)	0.03 (4.61)	− 16.88	14.28	853
GrimAge DNAmAA (years)	0.01 (3.08)	− 8.52	10.20	853
DunedinPACE (biol./chron.)	1.01 (0.11)	0.58	1.42	959

**Table 2:** Linear regression analysis of epigenetic age estimators on selenium status (deficient vs. sufficient) at baseline

Dependent variable	Model	St.* β*	*β*	SE	*p*	lowCI	upCI	*n*
Horvath clock DNAmAA	1	0.021	0.175	0.306	0.566	− 0.425	0.775	782
	2	0.019	0.155	0.320	0.628	− 0.473	0.783	684
GrimAge DNAmAA	1	0.010	0.065	0.222	0.771	− 0.371	0.500	782
	2	− 0.017	− 0.104	0.208	0.619	− 0.513	0.306	684
DunedinPACE	1	− 0.087	− 0.019	0.007	0.010	− 0.033	− 0.004	865
	2	− 0.087	− 0.019	0.007	0.012	− 0.034	− 0.004	757

Model 1 is unadjusted. Model 2 is adjusted for chronological age, sex, BMI, smoking (packyears), and the first four genetic principal components (PC1 to PC4)

St. = Standardized; SE = Standard Error; *p* = *p* value; lowCI = lower 95% confidence interval; upCI = upper 95% confidence interval

**Table 3:** Linear regression analysis of epigenetic age estimators on quartiles of SELENOP at baseline

Dependent Variable	Model		St.* β*	*β*	SE	*p*	lowCI	upCI	*n*
Horvath DNAmAA	1	Q2	0.033	0.310	0.435	0.477	− 0.545	1.164	765
		Q3	− 0.003	− 0.026	0.450	0.954	− 0.909	0.857	
		Q4	0.012	0.120	0.451	0.791	− 0.766	1.005	
	2	Q2	0.032	0.293	0.457	0.522	− 0.605	1.191	667
		Q3	0.026	0.252	0.467	0.590	− 0.666	1.169	
		Q4	0.007	0.067	0.476	0.888	− 0.867	1.001	
GrimAge DNAmAA	1	Q2	0.033	0.224	0.314	0.476	− 0.393	0.841	765
		Q3	− 0.071	− 0.514	0.325	0.114	− 1.152	0.124	
		Q4	0.004	0.028	0.326	0.932	− 0.612	0.667	
	2	Q2	0.032	0.220	0.298	0.460	− 0.365	0.806	667
		Q3	− 0.048	− 0.345	0.305	0.258	− 0.943	0.254	
		Q4	0.002	0.015	0.310	0.962	− 0.595	0.624	
DunedinPACE	1	Q2	− 0.076	− 0.018	0.010	0.078	− 0.039	0.002	848
		Q3	− 0.065	− 0.016	0.011	0.125	− 0.038	0.005	
		Q4	− 0.096	− 0.024	0.011	0.024	− 0.045	− 0.003	
	2	Q2	− 0.074	− 0.018	0.011	0.089	− 0.039	0.003	740
		Q3	− 0.067	− 0.017	0.011	0.119	− 0.038	0.004	
		Q4	− 0.115	− 0.030	0.011	0.007	− 0.051	− 0.008	

Model 1 is unadjusted. Model 2 is adjusted for chronological age, sex, BMI, smoking (packyears), and the first four genetic principal components (PC1 to PC4). The first quartile is used as reference

St. = Standardized; SE = Standard Error; *p* = *p* value; lowCI = lower 95% confidence interval; upCI = upper 95% confidence interval

**Table 4:** Linear regression analysis of epigenetic age estimators on quartiles of GPx3 intensity (proteomics data) at baseline

Dependent Variable	Model		St.* β*	*β*	SE	*p*	lowCI	upCI	*n*
Horvath DNAmAA	1	Q2	− 0.033	− 0.322	0.462	0.487	− 1.228	0.585	689
		Q3	− 0.076	− 0.772	0.471	0.102	− 1.697	0.153	
		Q4	− 0.064	− 0.628	0.458	0.171	− 1.527	0.272	
	2	Q2	− 0.016	− 0.151	0.478	0.753	− 1.090	0.788	608
		Q3	− 0.048	− 0.475	0.489	0.332	− 1.436	0.486	
		Q4	− 0.030	− 0.284	0.485	0.558	− 1.236	0.667	
GrimAge DNAmAA	1	Q2	0.004	0.026	0.332	0.937	− 0.625	0.677	689
		Q3	− 0.059	− 0.430	0.338	0.203	− 1.094	0.233	
		Q4	− 0.176	− 1.246	0.329	<0.001	− 1.892	− 0.601	
	2	Q2	− 0.044	− 0.313	0.310	0.313	− 0.923	0.296	608
		Q3	− 0.085	− 0.628	0.318	0.048	− 1.252	− 0.005	
		Q4	− 0.136	− 0.975	0.315	0.002	− 1.593	− 0.358	
DunedinPACE	1	Q2	− 0.006	− 0.002	0.011	0.885	− 0.023	0.020	764
		Q3	− 0.044	− 0.011	0.011	0.309	− 0.033	0.010	
		Q4	− 0.218	− 0.054	0.011	<0.001	− 0.076	− 0.033	
	2	Q2	− 0.011	− 0.003	0.011	0.801	− 0.024	0.019	674
		Q3	− 0.039	− 0.010	0.011	0.369	− 0.032	0.012	
		Q4	− 0.146	− 0.037	0.011	0.001	− 0.059	− 0.015	

Model 1 is unadjusted. Model 2 is adjusted for chronological age, sex, BMI, smoking (packyears), and the first four genetic principal components (PC1 to PC4). The first quartile is used as reference group

St. = Standardized; SE = Standard Error; *p* = *p* value; lowCI = lower 95% confidence interval; upCI = upper 95% confidence interval

### Assessment of selenium, SELENOP and GPx3

The selenium status biomarkers *SELENOP* and total selenium were measured at the Institute of Experimental Endocrinology, Charité University Hospital, Berlin, from serum samples that were taken during the same blood draw that produced the sample for the DNA isolation. Selenium concentration was measured by total reflection X-ray fluorescence (TXRF) spectroscopy as described in detail before [31, 32]. Selenium deficiency was defined as values below 90 μg/L. SELENOP was measured from serum samples with a sandwich ELISA method using monoclonal human antibodies. Additional information on how selenium and SELENOP were measured in BASE-II were previously described in detail [33]. GPx3 intensities at baseline were derived from proteomics data measured by liquid chromatography–mass spectrometry (LC–MS) using data-independent acquisition (Supplementary Methods).

### Confounders

Chronological age, sex, and smoking behavior (in packyears) were assessed during a one-on-one interview with trained study personnel. Body weight and height were measured with the electronic SECA measuring station (763, SECA, Germany). Genetic ancestry was controlled for by using the first four principal components from a principal component analysis on genome-wide SNP genotyping data. A detailed description of the procedures applied to calculate this confounder was published previously [34]. Leukocyte cell distribution was measured from samples obtained at the same blood-draw that was used for DNA isolation in an accredited clinical biochemistry laboratory (MVZ Labor 28 GmbH, Berlin, Germany) by automated standard methods (flow cytometry).

### Statistical analysis

Statistical analyses were conducted in R (version 4.3.1) [35]. Descriptive statistics were calculated with R’s tableone package [36]. Linear regression analyses were calculated with R’s *lm* and *lm.beta* function (lm.beta package [37]) to examine the relationship between DNAmA estimators (dependent variable) and selenium biomarkers (independent variables). Figures were drawn with the ggplot2 package. Selenium biomarker outliers were defined as < 25 th percentile − 1.5*SD and > the 75 th percentile + 1.5*SD. This resulted in the exclusion of *n* = 46 (serum selenium), *n* = 111 (SELENOP), and *n* = 151 (GPx3) observations. Participants with missing values in phenotypic data were excluded from the respective analyses (available case analyses). Linearity of the association between selenium biomarkers and epigenetic age estimators was assessed visually using scatterplots and using R’s geom_smooth function to fit a line to the plotted points. To assess possible nonlinear associations more objectively, the Ramsey Reset Test [38] using R’s resettest function (lmtest package) was performed (Supplementary Table 1). The results show statistical evidence for a nonlinear relationship between GrimAge and DunedinPACE with selenium/GPX3, and Horvath DNAmA with GPX3. While this supports the assumption of nonlinearity of these biomarker-clock-associations it does not necessarily mean that all other combinations are linear but only that we do not have enough evidence to conclude a putative nonlinearity based on the observed data. To facilitate the comparison of results between combinations of biomarker and selenium variables which are evidently nonlinear with the other combinations, the dichotomized selenium variables were used in the final analyses as this approach is valid for both linear and nonlinear associations. As a sensitivity analysis, all regression models were calculated using all available selenium biomarker values, i.e., without excluding outliers. Small nominal differences in the effect size estimates were observed, but did not change the interpretation of our results (Supplementary Tables 2, 3 and 4).

Statistical significance was defined at *α* = 0.05 to allow a better comparison of our results with that from previous work, who also used a nominal threshold to report significance. To allow a more differentiated view on the results of our study alone, we additionally applied a conservative multiple testing correction (using the Bonferroni method, adjusting for 18 tests, i.e., three selenium biomarkers × six epigenetic age measures) resulting in a study-wide *α* = 0.05/18 = 0.00278). Given the interdependence of both the selenium biomarkers and the epigenetic age measures, this adjustment is probably overly stringent. In line with the two other available studies analyzing the relationship between epigenetic age and selenium biomarkers [28, 39], we a priori decided to not adjust for multiple testing. Therefore, a cautious interpretation of the *p* value is needed, and results need to be externally verified.

We also analyzed the relationship between the individual GrimAge components and the selenium biomarkers (Supplementary Tables 5, 6, and 7). We do not consider these calculations to be part of our main analyses but included them in the supplementary material to enable a more detailed interpretation of the relationship between GrimAge DNAmAA and the selenium biomarkers.

This manuscript was prepared in accordance with the STROBE-nut reporting guidelines [40]. To prevent re-identification after matching of clinical variables across datasets from different BASE-II publications, it is unfortunately not possible to share BASE-II data publicly. However, sharing of data is possible under individual agreements. To get access to the data, interested researchers are encouraged to contact the BASE-II steering committee (Ludmila Müller, scientific coordinator, lmueller@mpib-berlin.mpg.de).

## Results

### Study population and characteristics

The sample analyzed in this study comprised 1568 BASE-II participants with a mean age of 69 years (SD = 3.4, range 60–85 years). Sex was almost equally distributed (51% women). 656 (48.3%) participants had a selenium level of 90 μg/L or lower, i.e., displaying selenium-deficiency. Study population characteristics are shown in Table 1.

### Association of selenium with biological age acceleration

Visual inspections of the distribution of serum selenium levels vs. DNAmAA estimated by the GrimAge and DunedinPACE clocks suggested possible nonlinear associations (Fig. 1). The Ramsey Reset Test revealed statistical evidence for a nonlinear relationship between GrimAge and selenium and between DunedinPACE and selenium (Supplementary Table 1). Therefore, in the next step we examined epigenetic age stratified by selenium deficiency (< 90 μg/L) and found a statistically significantly higher pace of aging measured by DunedinPACE in selenium-deficient participants (SMD = 0.2, *p* = 0.01, *t* test, Fig. 1F, Supplementary Table 8). DunedinPACE remained higher in selenium-deficient participants after the adjustment for known confounders (chronological age, sex, BMI, smoking, genetic ancestry) in linear regression analyses (*β* = − 0.02, SE = 0.007, 95% CI − 0.034 to − 0.004, *p* = 0.012, *n* = 757, Table 2). Sex-stratified analyses are shown in Supplementary Table 9 and show larger effect sizes in the subgroup of men compared to the subgroup of women.

### Association of SELENOP with biological age

Low SELENOP levels were similarly associated with accelerated GrimAge DNAmAA and DunedinPACE (Fig. 2). Assuming a nonlinear relationship based on the scatterplot (Fig. 2A–C), SELENOP values were categorized into quartiles. A statistically significantly higher pace of biological aging (DunedinPACE) was measured in participants of the lowest quartile compared to participants in the highest quartile (SMD = 0.21, *p* = 0.032, Fig. 2F). This association remained statistically significant after covariate adjustment (*β* = − 0.03, SE = 0.011, 95% CI − 0.051 to − 0.008, *p* = 0.007, Table 3, Supplementary Table 10). After further covariate adjustment the findings remained statistically significant in the subgroup of women (Supplementary Tables 11 and 12).

### Association of GPx3 with biological age

Finally, compared to the lowest quartile, participants in the fourth quartile had a higher age acceleration estimated from the GrimAge clock (SMD = 0.41, *p* = 0.0002) and DunedinPACE clock (SMD = 0.54, *p* = 2.2 × 10^− 7, Fig. 3). These associations persisted to be statistically significant after covariate adjustment (GrimAge: *β* = − 0.98, SE = 0.32, 95% CI − 1.59 to − 0.36, *p* = 0.002 and DunedinPACE: *β* = − 0.04, SE = 0.011, 95% CI − 0.06 to − 0.02, *p* = 0.001, Table 4, Supplementary Table 13). Sex-specific analyses are presented in Supplementary Tables 14 and 15.

## Discussion

In this study, we analyzed the association between serum biomarkers, namely total serum selenium, SELENOP, GPx3 and biological age measured by epigenetic clocks in 865 participants of BASE-II. Lower values in all three selenium biomarkers were associated with an increased pace of aging measured with the DunedinPACE clock. This association was independent from confounders included in the linear regression analysis.

The stepwise increase in the strength of association from the first- to the third-generation clock is likely due to the methodological approach used for their development with the later clocks potentially including CpG sites that better reflect the underlying aging process [39]. The first-generation clocks (e.g., 7-CpG, Horvath, and Hannum clock) were trained to predict chronological age. The subsequent generation of clocks (second-generation clocks, e.g., PhenoAge and GrimAge), however, focus on the training of biological aging measures. This comes with the advantage that information about the individual biological age is part of the epigenetic aging measure. This advantage of the second-generation clocks is also observable through a stronger association with the selenium biomarkers in this study. The latest development of the epigenetic clocks, the third-generation clock DunedinPACE algorithm, used longitudinal data for its training and represents the pace of aging similar to a speedometer (in contrast to the cumulative approach used for the first- and second-generation clocks which resembles an odometer). In our data, this clock shows the strongest association with the selenium biomarkers indicating that the longitudinal pace of aging is the most sensitive. This increased sensitivity toward exogenous effects [27] as well as toward clinical phenotypes was observed before [12–14, 41]. Interestingly, of the five individual CpGs (cg00163554, cg18212762, cg11270656, cg25194720, cg00886293) that were recently shown to be associated with urinary selenium levels in a large EWAS study [42] none was part of the aging algorithms analyzed here. This indicates that the selenium-associated differences in epigenetic aging most likely do not result from the change of individual CpGs but rather result from an overall difference in biological age measured as aggregate of all analyzed CpGs.

Our findings are in line with results published by Cheng and colleagues [27] who found a statistically significant association between serum selenium levels and DunedinPACE in a sample of 93 participants. In contrast, we could not validate the association between selenium levels and PhenoAge DNAmAA reported by the same authors (Supplementary Table 9). Furthermore, regarding our non-significant associations of selenium levels with earlier DNAmA clocks, our results are consistent with findings from a cohort of 276 older Chinese men and women that showed no statistically significant association between selenium and the first- and second-generation clocks [28].

Boyer and colleagues did not find a consistent association between urine selenium and any epigenetic age estimation in linear regression models in an American Indian population of 2301 participants (Strong Heart Study, SHS) [39]. However, when analyzing the effect of the total metal mixture using Bayesian Kernel Machine Regression, a nonlinear association with PhenoAge, GrimAge and DunedinPACE was found [39]. The difference in these results compared to our study may arise from the choice of method used to determine selenium status. Specifically, the selenium status in the SHS was replete [39], whereas BASE-II participants showed a borderline selenium-deficiency. The lack of effects observed in SHS may also be attributable to the saturated expression of selenoproteins, and thus likely reflecting a threshold effect as commonly observed for selenium and health outcomes.

Our analyses do not allow to draw any conclusions on cause-effect relationships between selenium levels and accelerated biological aging. However, our results corroborate recent findings on aging phenotypes assessed by other clinical and phenotypic outcomes that show an association between selenium biomarkers and mortality. In a recent prospective study with ~ 17 years of follow-up, serum concentrations of the selenium transporter SELENOP were inversely associated with all-cause and cardiovascular mortality, independent of biologically relevant confounders [22, 26, 43]. In line with this finding, serum selenium was inversely associated with mortality and incident heart failure in the Dutch PREVEND study comprising ~ 6000 individuals. Similar results were observed in other recent large prospective European studies for all-cause mortality as well as cardiovascular outcomes [23, 24, 43]. Besides all-cause mortality and cardiovascular outcomes, serum selenium biomarkers were shown to be inversely associated with prognosis in several cancer entities [21, 44–46]. Despite replete selenium status, similar associations have been observed in the NHANES study for mortality of different causes [26]. Moreover, an effect of selenium levels on epigenetic age is also biologically plausible since changes in the methylome in dependence to the selenium levels in rodents, cell-lines (human, mouse) and human tissue are well established [47]. Nevertheless, while clinical data in the literature, as well as our results indicate an association of selenium with improved health outcomes and healthy aging, the causality and involved mechanisms remain to be elucidated in future randomized controlled trials of sufficient size.

As noted in the results, only the association between GPx3 levels and both GrimAge and DunedinPACE, remain statistically significant after multiple testing correction, underscoring their likely relevance. Even if the other nominal associations do not withstand this correction, we note that the general findings are in agreement with the results from previous work, emphasizing the need to investigate the relationship between serum selenium and estimates of biological aging in larger datasets in future efforts.

Strengths of this study include the large sample size with an equal representation of men and women and a high degree of data completeness for the study parameters and potential confounders. Furthermore, this is the first study that examines additional selenium biomarkers, i.e., SELENOP and GPx3, in addition to selenium levels. The differing half-lives of these biomarkers ensure low risk of misclassification due to e.g., prior supplementation or enriched dietary selenium intake.

However, our study also has a few limitations. Firstly, as outlined above, the cross-sectional analyses presented in this study do not allow to draw any conclusions about causality or direction of effect. Secondly, the analyzed sample is of above-average health. Therefore, it is possible that the effects reported here are underestimated since BASE-II participants are overall healthier than the general population in Germany. In addition, this limits the generalizability of our results to the background population of mainly European elderly subjects with borderline sufficient selenium status residing in a metropolitan area. Thirdly, no detailed information about dosage and frequency of potential additional selenium intake by nutritional supplements was available. Therefore, all participants who reported selenium supplementation were excluded and evaluating effects of selenium supplementation on biological age was precluded. Finally, methylation data measured by the Illumina array was only available for the subgroup of participants who were part of the follow-up examination on average 7.4 years later. Due to the reduced study dataset as a result of study dropouts during the follow-up period, selection bias is introduced which, while we are confident it does not substantially impacts the results regarding the clocks analyzed here, prevents us from including mortality clocks, such as the Zhang clock [48], in our analysis.

## Conclusion

Our study shows that selenium biomarkers are moderately associated with accelerated biological aging in a European-based dataset. These results support previous findings that suggest health benefits of sufficient selenium levels. Randomized controlled trials are needed to evaluate the potential therapeutic benefit of selenium supplementation and its effect on biological age acceleration.

## Supplementary Information

Supplementary material 1.

Supplementary material 2.

## Data Availability

Due to concerns for participant privacy, data are available only upon reasonable request. Please contact Ludmila Müller, scientific coordinator, at lmueller@mpib-berlin.mpg.de, for additional information.
