# Supplementary material for: Low blood levels of selenium, selenoprotein P and GPx3 are associated with accelerated biological aging: results from the Berlin Aging Study II (BASE-II)
Source: Clin Epigenetics. 2025 Apr 25;17:62. doi: 10.1186/s13148-025-01863-7 (PMC12023433; doi:10.1186/s13148-025-01863-7)
Supplement: Supplementary file 1 — Supplementary material 1. [file 13148_2025_1863_MOESM1_ESM.pdf]

Low Blood Levels of Selenium, Selenoprotein P and GPx3 are Associated with Accelerated Biological Aging: Results from the Berlin Aging Study II (BASE-II)

Valentin Max Vetter [1,a], Kamil Demircan [2,a], Jan Homann [3], Thilo Samson Chillon [2], Michael Mülleder [5], Orr Shomroni [5], Elisabeth Steinhagen-Thiessen [1], Markus Ralser [5,6], Christina M. Lill [3, 4], Lars Bertram [7], Lutz Schomburg [2,b], Ilja Demuth [1,8,b]

[a] Valentin Max Vetter and Kamil Demircan are joint first authors.
[b] Lutz Schomburg and Ilja Demuth are joint last authors.

[1] Charité – Universitätsmedizin Berlin, corporate member of Freie Universität Berlin and Humboldt-Universität zu Berlin, Department of Endocrinology and Metabolic Diseases (including Division of Lipid Metabolism), Biology of Aging working group, Augustenburger Platz 1, 13353 Berlin, Germany

[2] Institute for Experimental Endocrinology, Charité - Universitätsmedizin Berlin, Corporate Member of Freie Universität Berlin and Humboldt-Universität zu Berlin, Max Rubner Center (MRC) for Cardiovascular Metabolic Renal Research, D-10115, Berlin, Germany

[3] Institute of Epidemiology and Social Medicine, University of Münster, Münster, Germany

[4] Ageing Epidemiology Research Unit (AGE), School of Public Health, Imperial College London, London, UK

[5] Core Facility High Throughput Mass Spectrometry, Charité - Universitätsmedizin Berlin, corporate member of Freie Universität Berlin and Humboldt-Universität zu Berlin, Berlin, Germany

[6] The Centre for Human Genetics, Nuffield Department of Medicine, University of Oxford, UK

[7] Lübeck Interdisciplinary Platform for Genome Analytics (LIGA), University of Lübeck, Lübeck, Germany

[8] Berlin Institute of Health at Charité – Universitätsmedizin Berlin, BCRT – Berlin Institute of Health Center for Regenerative Therapies, Berlin, Germany

Corresponding Author:
Ilja Demuth (Ph.D.)
Charité - Universitätsmedizin Berlin
Lipid Clinic at the Interdisciplinary Metabolism Center,
Biology of Aging Group
Augustenburger Platz 1
13353 Berlin
Email: ilja.demuth@charite.de
Phone: ++49 30 450 569 143
FAX: ++49 30 450 566 904
ORCID: https://orcid.org/0000-0002-4340-2523

Valentin Max Vetter (MD, M.Sc.)
Charité - Universitätsmedizin Berlin
Lipid Clinic at the Interdisciplinary Metabolism Center,
Biology of Aging Group
Augustenburger Platz 1
13353 Berlin
Email: valentin.vetter@charite.de
Phone: ++49 30 450 566 298
ORCID: https://orcid.org/0000-0001-5003-7766

Supplementary Material

Methods:

Measurement of GPx3

The sample preparation involved semi-automated processing of serum samples in 96-well plates, including denaturation, reduction, alkylation, trypsin digestion, and cleanup steps (34). Liquid chromatography-mass spectrometry (LC-MS) analysis was conducted using a Bruker timsTOF Pro system coupled with an Agilent 1290 Infinity II LC system (35). Computational proteomics involved generating a spectral library based on Human Plasma PeptideAtlas and annotating peptide sequences to the Uniprot human reference proteome (36, 37). The software DIA-NN was used for data annotation and quantification (38). Pre-processing was performed using MS-DAP (39) as framework and included data normalization, outlier sample filtering, low-presence peptide filtering, data imputation, and batch correction to ensure high data quality.

Tables:

Supplementary Table 1: Ramsey Reset test of a linear regression model.

Dependent Variable	Independent Variable	p-value
Horvath DNAmAA	Selenium (Serum)	0.555
GrimAge DNAmAA	Selenium (Serum)	0.018
DunedinPACE	Selenium (Serum)	0.043
Horvath DNAmAA	SELENOP	0.980
GrimAge DNAmAA	SELENOP	0.394
DunedinPACE	SELENOP	0.490
Horvath DNAmAA	GPX3	0.031
GrimAge DNAmAA	GPX3	0.594
DunedinPACE	GPX3	0.046

Note: DNAmAA = DNA methylation age acceleration.

Supplementary Table 2: Linear regression analysis of epigenetic age estimators on selenium status (deficient vs. sufficient) at baseline **including all outliers**. Model 1 is unadjusted. Model 2 is adjusted for chronological age, sex, BMI, smoking (packyears), and the first four genetic principal components (PC1 to PC4).

Dependent Variable	Model	St. β	β	SE	p	lowCI	upCI	n
Horvath Clock	1	0.017	0.143	0.301	0.635	-0.447	0.733	804
	2	0.013	0.105	0.314	0.739	-0.511	0.721	704
GrimAge	1	0.017	0.102	0.218	0.639	-0.326	0.531	804
	2	-0.015	-0.092	0.205	0.652	-0.495	0.310	704
DunedinPACE	1	-0.086	-0.018	0.007	0.011	-0.033	-0.004	889
	2	-0.087	-0.019	0.007	0.010	-0.033	-0.005	779

Supplementary Table 3: Linear regression analysis of epigenetic age estimators on quartiles of SELENOP at baseline. Model 1 is unadjusted **including all outliers**. Model 2 is adjusted for chronological age, sex, BMI, smoking (packyears), and the first four genetic principal components (PC1 to PC4). The first quartile is used as reference.

Dependent Variable	Model		St. β	β	SE	p	lowCI	upCI	n
Horvath DNAmAA	1	Q2	0.039	0.373	0.416	0.371	-0.444	1.190	822
		Q3	0.032	0.317	0.430	0.460	-0.526	1.161	822
		Q4	0.003	0.031	0.431	0.943	-0.815	0.876	822
	2	Q2	0.045	0.423	0.436	0.332	-0.433	1.279	720
		Q3	0.052	0.501	0.446	0.261	-0.375	1.377	720
		Q4	0.004	0.040	0.451	0.929	-0.846	0.926	720
GrimAge	1	Q2	0.020	0.141	0.301	0.640	-0.450	0.732	822
		Q3	-0.049	-0.353	0.311	0.256	-0.963	0.257	822
		Q4	0.004	0.029	0.311	0.926	-0.582	0.640	822
	2	Q2	0.044	0.309	0.282	0.275	-0.246	0.863	720
		Q3	-0.020	-0.141	0.289	0.625	-0.708	0.426	720
		Q4	0.008	0.056	0.292	0.849	-0.518	0.629	720
DunedinPACE	1	Q2	-0.072	-0.017	0.010	0.080	-0.037	0.002	912
		Q3	-0.070	-0.017	0.010	0.087	-0.037	0.002	912
		Q4	-0.087	-0.022	0.010	0.032	-0.042	-0.002	912
	2	Q2	-0.053	-0.013	0.010	0.197	-0.033	0.007	800
		Q3	-0.058	-0.014	0.010	0.154	-0.034	0.005	800
		Q4	-0.096	-0.025	0.010	0.018	-0.045	-0.004	800

Supplementary Table 4: Linear regression analysis of epigenetic age estimators on quartiles of GPx3 intensity (proteomics data) at baseline **including all outliers**. Model 1 is unadjusted. Model 2 is adjusted for chronological age, sex, BMI, smoking (packyears), and the first four genetic principal components (PC1 to PC4). The first quartile is used as reference group.

Dependent Variable	Model		St. *β*	*β*	SE	p	lowCI	upCI	n
Horvath DNAmAA	1	Q2	-0.024	-0.233	0.438	0.595	-1.094	0.627	759
		Q3	-0.075	-0.745	0.444	0.094	-1.616	0.126	759
		Q4	-0.066	-0.645	0.435	0.138	-1.498	0.208	759
	2	Q2	-0.002	-0.023	0.453	0.960	-0.912	0.867	670
		Q3	-0.043	-0.416	0.458	0.364	-1.315	0.483	670
		Q4	-0.037	-0.352	0.460	0.445	-1.254	0.551	670
GrimAge	1	Q2	-0.017	-0.125	0.317	0.693	-0.746	0.497	759
		Q3	-0.067	-0.490	0.321	0.127	-1.119	0.139	759
		Q4	-0.166	-1.178	0.314	<0.001	-1.795	-0.562	759
	2	Q2	-0.048	-0.349	0.295	0.237	-0.927	0.230	670
		Q3	-0.076	-0.557	0.298	0.062	-1.142	0.027	670
		Q4	-0.134	-0.963	0.299	0.001	-1.550	-0.375	670
DunedinPACE	1	Q2	-0.052	-0.013	0.010	0.213	-0.033	0.007	845
		Q3	-0.068	-0.017	0.010	0.100	-0.038	0.003	845
		Q4	-0.233	-0.058	0.010	<0.001	-0.078	-0.038	845
	2	Q2	-0.056	-0.014	0.010	0.174	-0.035	0.006	746
		Q3	-0.056	-0.014	0.010	0.175	-0.035	0.006	746
		Q4	-0.158	-0.040	0.011	<0.001	-0.061	-0.019	746

Supplementary Table 5: Linear regression analysis of individual GrimAge components on selenium status (deficient vs. sufficient) at baseline. Model 1 is unadjusted. Model 2 is adjusted for chronological age, sex, BMI, smoking (packyears), and the first four genetic principal components (PC1 to PC4).

Dependent Variable	Model	St. β	β	SE	p	lowCI	upCI	n
DNAmADM	1	-0.053	-1.847	1.248	0.139	-4.296	0.602	782
	2	-0.009	-0.331	1.109	0.766	-2.509	1.847	684
DNAmB2M	1	-0.016	-2794.802	6094.793	0.647	-14758.941	9169.338	782
	2	0.000	23.919	5428.772	0.996	-10635.420	10683.259	684
DNAmCystatinC	1	-0.006	-268.134	1701.126	0.875	-3607.462	3071.193	782
	2	0.016	789.333	1635.596	0.630	-2422.142	4000.809	684
DNAmGDF15	1	-0.052	-15.661	10.726	0.145	-36.716	5.394	782
	2	-0.058	-17.768	11.295	0.116	-39.945	4.409	684
DNAmLeptin	1	-0.031	-236.017	276.467	0.394	-778.725	306.692	782
	2	0.006	45.760	199.506	0.819	-345.968	437.488	684
DNAmPACKYRS	1	0.014	0.225	0.591	0.704	-0.936	1.385	782
	2	-0.016	-0.266	0.539	0.622	-1.324	0.793	684
DNAmPAI1	1	0.007	31.619	171.002	0.853	-304.060	367.298	782
	2	-0.003	-14.462	162.828	0.929	-334.173	305.250	684
DNAmTIMP1	1	-0.075	-106.375	50.372	0.035	-205.257	-7.493	782
	2	-0.047	-67.298	32.571	0.039	-131.251	-3.345	684

Note: St. = Standardized; SE = Standard Error; p = p-value; lowCI = lower 95% confidence interval; upCI = upper 95% confidence interval, ADM = adrenomedullin, B2M = beta-2-microglobulim, GDF15 = growth differentiation factor 15, PAI1 = plasminogen activator inhibitor 1, TIMP1 = tissue Inhibitor Metalloproteinases 1.

Supplementary Table 6: Linear regression analysis of individual GrimAge components on quartiles of SELENOP at baseline. Model 1 is unadjusted. Model 2 is adjusted for chronological age, sex, BMI, smoking (packyears), and the first four genetic principal components (PC1 to PC4). The first quartile is used as reference.

Dependent Variable	Model		St. β	β	SE	p	lowCI	upCI	n
DNAmADM	1	Q2	-0.012	-0.464	1.787	0.795	-3.971	3.043	765
		Q3	-0.010	-0.399	1.847	0.829	-4.024	3.227	765
		Q4	-0.028	-1.137	1.852	0.539	-4.772	2.498	765
	2	Q2	0.030	1.182	1.590	0.457	-1.939	4.303	667
		Q3	-0.014	-0.561	1.624	0.730	-3.749	2.628	667
		Q4	0.029	1.194	1.654	0.470	-2.053	4.441	667
DNAmB2M	1	Q2	-0.001	-181.188	8663.866	0.983	-17189.103	16826.727	765
		Q3	0.009	1754.600	8956.299	0.845	-15827.386	19336.586	765
		Q4	-0.039	-7772.593	8979.414	0.387	-25399.956	9854.770	765
	2	Q2	0.038	7121.426	7765.657	0.359	-8127.160	22370.011	667
		Q3	0.003	524.866	7933.120	0.947	-15052.548	16102.280	667
		Q4	0.004	800.708	8078.836	0.921	-15062.832	16664.248	667
DNAmCystatinC	1	Q2	0.021	1125.090	2429.670	0.643	-3644.561	5894.742	765
		Q3	0.042	2338.056	2511.679	0.352	-2592.586	7268.699	765
		Q4	0.015	824.981	2518.161	0.743	-4118.386	5768.349	765
	2	Q2	0.041	2177.813	2361.137	0.357	-2458.498	6814.125	667
		Q3	0.032	1772.938	2412.054	0.463	-2963.353	6509.229	667
		Q4	0.040	2270.434	2456.359	0.356	-2552.853	7093.722	667
DNAmGDF15	1	Q2	-0.035	-11.657	15.366	0.448	-41.822	18.508	765
		Q3	-0.016	-5.750	15.885	0.717	-36.933	25.433	765
		Q4	-0.070	-24.723	15.926	0.121	-55.986	6.541	765
	2	Q2	0.008	2.817	16.339	0.863	-29.267	34.901	667
		Q3	-0.021	-7.347	16.692	0.660	-40.123	25.429	667
		Q4	-0.055	-19.961	16.998	0.241	-53.339	13.417	667
DNAmLeptin	1	Q2	0.008	72.244	393.799	0.854	-700.817	845.305	765
		Q3	0.031	280.796	407.091	0.491	-518.358	1079.950	765
		Q4	-0.008	-72.270	408.141	0.859	-873.487	728.946	765
	2	Q2	0.033	287.736	286.139	0.315	-274.124	849.596	667
		Q3	0.013	117.444	292.309	0.688	-456.532	691.421	667
		Q4	0.016	148.643	297.678	0.618	-435.877	733.162	667
DNAmPACKYRS	1	Q2	-0.030	-0.553	0.836	0.509	-2.195	1.089	765
		Q3	-0.088	-1.687	0.865	0.051	-3.384	0.010	765
		Q4	-0.059	-1.126	0.867	0.194	-2.827	0.576	765
	2	Q2	-0.035	-0.640	0.769	0.405	-2.151	0.870	667
		Q3	-0.077	-1.465	0.786	0.063	-3.007	0.078	667
		Q4	-0.049	-0.959	0.800	0.231	-2.530	0.612	667
DNAmPAI1	1	Q2	0.027	142.563	244.007	0.559	-336.442	621.569	765
		Q3	0.011	60.439	252.243	0.811	-434.735	555.613	765
		Q4	0.040	221.561	252.894	0.381	-274.890	718.013	765
	2	Q2	0.037	196.011	233.179	0.401	-261.859	653.880	667

		Q3	0.007	36.214	238.208	0.879	-431.529	503.957	667
		Q4	0.009	49.453	242.583	0.839	-426.881	525.787	667
DNAmTIMP1	1	Q2	-0.019	-30.006	71.666	0.676	-170.692	110.681	765
		Q3	0.039	64.021	74.085	0.388	-81.414	209.455	765
		Q4	-0.052	-85.114	74.276	0.252	-230.924	60.696	765
	2	Q2	0.023	36.129	46.827	0.441	-55.820	128.078	667
		Q3	0.007	11.130	47.837	0.816	-82.802	105.062	667
		Q4	-0.023	-38.993	48.715	0.424	-134.650	56.664	667

Note: St. = Standardized; SE = Standard Error; p = p-value; lowCI = lower 95% confidence interval; upCI = upper 95% confidence interval, ADM = adrenomedullin, B2M = beta-2-microglobulim, GDF15 = growth differentiation factor 15, PAI1 = plasminogen activator inhibitor 1, TIMP1 = tissue Inhibitor Metalloproteinases 1.

Supplementary Table 7: Linear regression analysis of individual GrimAge components on quartiles of GPx3 intensity at baseline. Model 1 is unadjusted. Model 2 is adjusted for chronological age, sex, BMI, smoking (packyears), and the first four genetic principal components (PC1 to PC4). The first quartile is used as reference group.

Dependent Variable	Model		St. β	β	SE	p	lowCI	upCI	n
DNAmADM	1	Q2	0.018	0.749	1.917	0.696	-3.014	4.512	689
		Q3	-0.066	-2.777	1.954	0.156	-6.614	1.060	689
		Q4	0.006	0.252	1.901	0.895	-3.481	3.984	689
	2	Q2	0.007	0.283	1.680	0.866	-3.016	3.582	608
		Q3	-0.056	-2.360	1.718	0.170	-5.735	1.015	608
		Q4	-0.008	-0.331	1.702	0.846	-3.673	3.011	608
DNAmB2M	1	Q2	-0.043	-8.395.904	9.200.499	0.362	-26.460.469	9.668.660	689
		Q3	-0.085	-17.205.241	9.381.169	0.067	-35.624.540	1.214.057	689
		Q4	-0.099	-19.265.033	9.124.822	0.035	-37.181.011	-1.349.055	689
	2	Q2	-0.032	-6.385.469	8.193.027	0.436	-22.476.183	9.705.244	608
		Q3	-0.064	-12.945.491	8.380.657	0.123	-29.404.701	3.513.718	608
		Q4	-0.055	-10.781.826	8.300.706	0.194	-27.084.017	5.520.364	608
DNAmCystatinC	1	Q2	-0.037	-2.005.129	2.498.774	0.423	-6.911.305	2.901.046	689
		Q3	-0.096	-5.298.463	2.547.842	0.038	-10.300.981	-295.945	689
		Q4	-0.121	-6.416.335	2.478.221	0.010	-11.282.155	-1.550.514	689
	2	Q2	-0.053	-2.826.058	2.381.538	0.236	-7.503.284	1.851.168	608
		Q3	-0.106	-5.793.271	2.436.077	0.018	-10.577.611	-1.008.932	608
		Q4	-0.103	-5.489.951	2.412.838	0.023	-10.228.649	-751.254	608
DNAmGDF15	1	Q2	-0.019	-6.525	16.010	0.684	-37.960	24.910	689
		Q3	-0.071	-24.946	16.325	0.127	-56.999	7.106	689
		Q4	-0.062	-20.875	15.879	0.189	-52.051	10.302	689
	2	Q2	-0.045	-15.522	16.760	0.355	-48.438	17.393	608
		Q3	-0.072	-25.784	17.144	0.133	-59.453	7.885	608
		Q4	-0.070	-24.093	16.980	0.156	-57.441	9.255	608
DNAmLeptin	1	Q2	-0.035	-315.378	418.831	0.452	-1.137.725	506.970	689
		Q3	-0.043	-392.780	427.056	0.358	-1.231.275	445.716	689
		Q4	0.070	621.056	415.386	0.135	-194.527	1.436.639	689
	2	Q2	-0.040	-366.902	294.074	0.213	-944.449	210.644	608
		Q3	-0.038	-359.407	300.808	0.233	-950.180	231.366	608
		Q4	-0.019	-170.261	297.939	0.568	-755.398	414.876	608
DNAmPACKYRS	1	Q2	0.018	0.336	0.885	0.704	-1.402	2.074	689
		Q3	-0.026	-0.513	0.903	0.570	-2.285	1.260	689
		Q4	-0.153	-2.895	0.878	0.001	-4.619	-1.171	689
	2	Q2	-0.029	-0.561	0.800	0.483	-2.132	1.010	608
		Q3	-0.059	-1.163	0.818	0.156	-2.770	0.445	608
		Q4	-0.131	-2.510	0.811	0.002	-4.102	-0.918	608
DNAmPAI1	1	Q2	-0.018	-95.749	248.040	0.700	-582.759	391.261	689
		Q3	-0.075	-413.133	252.911	0.103	-909.707	83.440	689
		Q4	-0.200	-1.062.057	246.000	0.00002	-1.545.062	-579.053	689
	2	Q2	-0.002	-8.303	237.362	0.972	-474.471	457.866	608

		Q3	-0.036	-201.773	242.798	0.406	-678.617	275.072	608
		Q4	-0.085	-458.484	240.482	0.057	-930.780	13.811	608
DNAmTIMP1	1	Q2	-0.028	-45.169	76.134	0.553	-194.653	104.315	689
		Q3	-0.056	-94.651	77.629	0.223	-247.071	57.768	689
		Q4	-0.159	-258.192	75.508	0.001	-406.446	-109.937	689
	2	Q2	-0.031	-50.490	48.764	0.301	-146.259	45.280	608
		Q3	-0.034	-56.771	49.880	0.256	-154.733	41.191	608
		Q4	-0.062	-101.293	49.404	0.041	-198.321	-4.265	608

Note: St. = Standardized; SE = Standard Error; p = p-value; lowCI = lower 95% confidence interval; upCI = upper 95% confidence interval, ADM = adrenomedullin, B2M = beta-2-microglobulim, GDF15 = growth differentiation factor 15, PAI1 = plasminogen activator inhibitor 1, TIMP1 = tissue Inhibitor Metalloproteinases 1.

Supplementary Table 8: Standardized mean difference of epigenetic age estimators between selenium deficient participants and selenium sufficient participants. Statistical significance of difference was assessed by t-test.

Variable	Deficient Mean(SD)	n	Sufficient Mean (SD)	n	p	SMD
Horvath DNAmAA	-0.03 (4.20)	391	0.15 (4.34)	391	0.566	0.04
GrimAge DNAmAA	-0.11 (3.08)	391	-0.04 (3.13)	391	0.771	0.02
DunedinPACE	1.02 (0.11)	440	1.00 (0.10)	425	0.010	0.18

Note: SD = Standard Deviation, n = number of observations, SMD = Standardized Mean Difference.

Supplementary Table 9: Linear regression analysis of epigenetic age estimators calculated from all six epigenetic clocks available on selenium status (deficient vs. sufficient) in the complete dataset as well as sex-stratified subgroups. Model 1 is unadjusted. Model 2 is adjusted for chronological age, sex, BMI, smoking (packyears), and the first four genetic principal components (PC1 to PC4).

Clock	Model	St. β	β	SE	p	lowCI	upCI	n
Women and Men								
7-CpG DNAmAA	1	0.022	0.298	0.396	0.452	-0.480	1.075	1198
	2	0.004	0.058	0.521	0.912	-0.965	1.081	691
Horvath DNAmAA	1	0.021	0.175	0.306	0.566	-0.425	0.775	782
	2	0.019	0.155	0.320	0.628	-0.473	0.783	684
Hannum DNAmAA	1	0.002	0.015	0.250	0.953	-0.475	0.505	782
	2	0.002	0.015	0.262	0.955	-0.499	0.529	684
PhenoAge DNAmAA	1	-0.036	-0.330	0.332	0.320	-0.983	0.322	782
	2	-0.038	-0.344	0.352	0.328	-1.034	0.346	684
GrimAge DNAmAA	1	0.010	0.065	0.222	0.771	-0.371	0.500	782
	2	-0.017	-0.104	0.208	0.619	-0.513	0.306	684
DunedinPACE	1	-0.087	-0.019	0.007	0.010	-0.033	-0.004	865
	2	-0.087	-0.019	0.007	0.012	-0.034	-0.004	757
Women								
7-CpG DNAmAA	1	0.054	0.713	0.542	0.189	-0.352	1.778	602
	2	0.088	1.185	0.725	0.103	-0.241	2.612	357
Horvath DNAmAA	1	0.025	0.205	0.415	0.621	-0.611	1.021	400
	2	0.041	0.340	0.446	0.446	-0.537	1.218	354
Hannum DNAmAA	1	-0.024	-0.155	0.327	0.637	-0.798	0.489	400
	2	0.001	0.007	0.350	0.984	-0.682	0.695	354
PhenoAge DNAmAA	1	-0.051	-0.476	0.468	0.310	-1.396	0.444	400
	2	-0.055	-0.518	0.499	0.300	-1.500	0.464	354
GrimAge DNAmAA	1	0.018	0.103	0.278	0.713	-0.444	0.649	400
	2	-0.005	-0.027	0.288	0.924	-0.594	0.539	354
DunedinPACE	1	-0.083	-0.017	0.010	0.081	-0.035	0.002	447
	2	-0.066	-0.013	0.010	0.176	-0.032	0.006	395
Men								
7-CpG DNAmAA	1	-0.018	-0.241	0.566	0.670	-1.353	0.870	596
	2	-0.078	-1.062	0.752	0.159	-2.542	0.418	334
Horvath DNAmAA	1	0.001	0.007	0.439	0.987	-0.856	0.871	382
	2	-0.009	-0.077	0.465	0.869	-0.992	0.838	330
Hannum DNAmAA	1	0.007	0.051	0.364	0.889	-0.665	0.767	382
	2	-0.006	-0.044	0.396	0.912	-0.823	0.735	330
PhenoAge DNAmAA	1	-0.027	-0.246	0.471	0.602	-1.172	0.680	382
	2	-0.022	-0.195	0.500	0.697	-1.178	0.788	330
GrimAge DNAmAA	1	-0.031	-0.184	0.309	0.552	-0.793	0.424	382
	2	-0.030	-0.185	0.305	0.546	-0.786	0.416	330
DunedinPACE	1	-0.119	-0.026	0.011	0.015	-0.047	-0.005	418
	2	-0.109	-0.024	0.011	0.034	-0.047	-0.002	362

Note: St. = Standardized; SE = Standard Error; p = p-value; lowCI = lower 95% confidence interval; upCI = upper 95% confidence interval.

Supplementary Table 10: Linear regression analysis of epigenetic age estimates calculated by all available six epigenetic clocks on quartiles of Selenoprotein P in men and women. Model 1 is unadjusted. Model 2 is adjusted for chronological age, sex, BMI, smoking (packyears), and the first four genetic principal components (PC1 to PC4). The first quartile is used as reference.

Clock	Model		St. β	β	SE	p	lowCI	upCI	n
7-CpG DNAmAA	1	Q2	-0.018	-0.284	0.563	0.613	-1.388	0.820	1168
		Q3	-0.033	-0.525	0.567	0.354	-1.637	0.587	1168
		Q4	0.007	0.109	0.568	0.847	-1.005	1.224	1168
	2	Q2	0.051	0.771	0.734	0.294	-0.671	2.213	674
		Q3	-0.001	-0.020	0.753	0.979	-1.497	1.458	674
		Q4	0.022	0.348	0.764	0.649	-1.152	1.848	674
Horvath DNAmAA	1	Q2	0.033	0.310	0.435	0.477	-0.545	1.164	765
		Q3	-0.003	-0.026	0.450	0.954	-0.909	0.857	765
		Q4	0.012	0.120	0.451	0.791	-0.766	1.005	765
	2	Q2	0.032	0.293	0.457	0.522	-0.605	1.191	667
		Q3	0.026	0.252	0.467	0.590	-0.666	1.169	667
		Q4	0.007	0.067	0.476	0.888	-0.867	1.001	667
Hannum DNAmAA	1	Q2	0.111	0.857	0.354	0.016	0.162	1.551	765
		Q3	-0.023	-0.184	0.366	0.616	-0.901	0.534	765
		Q4	0.004	0.031	0.367	0.932	-0.688	0.751	765
	2	Q2	0.132	1.002	0.372	0.007	0.273	1.732	667
		Q3	0.011	0.086	0.380	0.821	-0.659	0.831	667
		Q4	0.012	0.098	0.386	0.801	-0.661	0.857	667
PhenoAge DNAmAA	1	Q2	0.024	0.252	0.475	0.596	-0.681	1.184	765
		Q3	-0.024	-0.255	0.491	0.604	-1.219	0.709	765
		Q4	-0.016	-0.177	0.492	0.719	-1.144	0.790	765
	2	Q2	0.026	0.269	0.505	0.594	-0.722	1.261	667
		Q3	-0.027	-0.287	0.516	0.578	-1.300	0.726	667
		Q4	-0.039	-0.417	0.525	0.427	-1.449	0.614	667
GrimAge DNAmAA	1	Q2	0.033	0.224	0.314	0.476	-0.393	0.841	765
		Q3	-0.071	-0.514	0.325	0.114	-1.152	0.124	765
		Q4	0.004	0.028	0.326	0.932	-0.612	0.667	765
	2	Q2	0.032	0.220	0.298	0.460	-0.365	0.806	667
		Q3	-0.048	-0.345	0.305	0.258	-0.943	0.254	667
		Q4	0.002	0.015	0.310	0.962	-0.595	0.624	667
DunedinPACE	1	Q2	-0.076	-0.018	0.010	0.078	-0.039	0.002	848
		Q3	-0.065	-0.016	0.011	0.125	-0.038	0.005	848
		Q4	-0.096	-0.024	0.011	0.024	-0.045	-0.003	848
	2	Q2	-0.074	-0.018	0.011	0.089	-0.039	0.003	740
		Q3	-0.067	-0.017	0.011	0.119	-0.038	0.004	740
		Q4	-0.115	-0.030	0.011	0.007	-0.051	-0.008	740

Note: St. = Standardized; SE = Standard Error; p = p-value; lowCI = lower 95% confidence interval; upCI = upper 95% confidence interval.

Supplementary Table 11: Linear regression analysis of epigenetic age estimates calculated by all available six epigenetic clocks on quartiles of Selenoprotein P in the subgroup of women. Model 1 is unadjusted. Model 2 is adjusted for chronological age, sex, BMI, smoking (packyears), and the first four genetic principal components (PC1 to PC4). The first quartile is used as reference.

Clock	Model		St. β	β	SE	p	lowCI	upCI	n
7-CpG DNAmAA	1	Q2	0.027	0.407	0.760	0.593	-1.085	1.898	588
		Q3	0.018	0.265	0.764	0.729	-1.236	1.766	588
		Q4	0.084	1.272	0.772	0.100	-0.245	2.789	588
	2	Q2	0.126	1.815	1.002	0.071	-0.156	3.785	348
		Q3	0.025	0.365	1.015	0.719	-1.631	2.361	348
		Q4	0.044	0.688	1.069	0.520	-1.414	2.791	348
Horvath DNAmAA	1	Q2	0.096	0.877	0.589	0.137	-0.281	2.036	391
		Q3	0.083	0.786	0.603	0.194	-0.400	1.972	391
		Q4	0.061	0.603	0.622	0.333	-0.620	1.826	391
	2	Q2	0.045	0.412	0.633	0.516	-0.833	1.657	345
		Q3	0.072	0.669	0.638	0.295	-0.586	1.925	345
		Q4	0.035	0.345	0.675	0.609	-0.982	1.673	345
Hannum DNAmAA	1	Q2	0.137	0.993	0.463	0.033	0.083	1.904	391
		Q3	0.036	0.271	0.474	0.568	-0.661	1.203	391
		Q4	0.021	0.165	0.489	0.736	-0.796	1.126	391
	2	Q2	0.163	1.162	0.493	0.019	0.192	2.131	345
		Q3	0.050	0.362	0.497	0.466	-0.615	1.340	345
		Q4	0.025	0.192	0.525	0.716	-0.842	1.225	345
PhenoAge DNAmAA	1	Q2	0.075	0.772	0.665	0.247	-0.536	2.080	391
		Q3	0.006	0.069	0.681	0.920	-1.271	1.408	391
		Q4	0.037	0.418	0.703	0.552	-0.964	1.799	391
	2	Q2	0.055	0.567	0.709	0.424	-0.827	1.962	345
		Q3	-0.005	-0.053	0.715	0.941	-1.459	1.353	345
		Q4	0.011	0.129	0.756	0.865	-1.358	1.616	345
GrimAge DNAmAA	1	Q2	0.054	0.329	0.394	0.405	-0.446	1.103	391
		Q3	-0.068	-0.433	0.403	0.284	-1.226	0.360	391
		Q4	-0.001	-0.009	0.416	0.984	-0.826	0.809	391
	2	Q2	0.051	0.319	0.407	0.434	-0.482	1.120	345
		Q3	-0.079	-0.505	0.411	0.219	-1.313	0.303	345
		Q4	-0.016	-0.112	0.434	0.796	-0.966	0.742	345
DunedinPACE	1	Q2	-0.101	-0.023	0.014	0.095	-0.049	0.004	438
		Q3	-0.058	-0.013	0.014	0.335	-0.040	0.014	438
		Q4	-0.108	-0.026	0.014	0.068	-0.054	0.002	438
	2	Q2	-0.086	-0.019	0.014	0.161	-0.046	0.008	386
		Q3	-0.095	-0.022	0.014	0.119	-0.049	0.006	386
		Q4	-0.133	-0.032	0.015	0.028	-0.061	-0.004	386

Note: St. = Standardized; SE = Standard Error; p = p-value; lowCI = lower 95% confidence interval; upCI = upper 95% confidence interval.

Supplementary Table 12: Linear regression analysis of epigenetic age estimates calculated by all available six epigenetic clocks on quartiles of Selenoprotein P in the subgroup of men. Model 1 is unadjusted. Model 2 is adjusted for chronological age, sex, BMI, smoking (packyears), and the first four genetic principal components (PC1 to PC4). The first quartile is used as reference.

Clock	Model		St. β	β	SE	p	lowCI	upCI	n
7-CpG DNAmAA	1	Q2	-0.054	-0.865	0.811	0.286	-2.458	0.727	580
		Q3	-0.074	-1.202	0.818	0.142	-2.809	0.405	580
		Q4	-0.062	-1.002	0.812	0.218	-2.597	0.594	580
	2	Q2	-0.017	-0.261	1.097	0.812	-2.418	1.897	326
		Q3	-0.033	-0.527	1.137	0.643	-2.764	1.709	326
		Q4	0.010	0.153	1.111	0.890	-2.033	2.340	326
Horvath DNAmAA	1	Q2	-0.029	-0.272	0.623	0.663	-1.498	0.954	374
		Q3	-0.082	-0.829	0.652	0.204	-2.110	0.452	374
		Q4	-0.050	-0.482	0.635	0.449	-1.730	0.767	374
	2	Q2	0.011	0.102	0.677	0.880	-1.230	1.433	322
		Q3	-0.037	-0.358	0.701	0.610	-1.736	1.021	322
		Q4	-0.033	-0.312	0.686	0.649	-1.663	1.038	322
Hannum DNAmAA	1	Q2	0.093	0.730	0.513	0.156	-0.279	1.739	374
		Q3	-0.072	-0.601	0.536	0.263	-1.656	0.453	374
		Q4	-0.027	-0.216	0.522	0.679	-1.244	0.811	374
	2	Q2	0.113	0.882	0.571	0.124	-0.242	2.007	322
		Q3	-0.023	-0.192	0.592	0.746	-1.356	0.972	322
		Q4	-0.018	-0.146	0.579	0.802	-1.286	0.994	322
PhenoAge DNAmAA	1	Q2	-0.028	-0.288	0.675	0.670	-1.616	1.040	374
		Q3	-0.050	-0.544	0.706	0.441	-1.932	0.844	374
		Q4	-0.078	-0.820	0.688	0.234	-2.172	0.532	374
	2	Q2	-0.005	-0.046	0.733	0.950	-1.488	1.395	322
		Q3	-0.058	-0.611	0.758	0.421	-2.103	0.881	322
		Q4	-0.100	-1.039	0.743	0.163	-2.501	0.422	322
GrimAge DNAmAA	1	Q2	0.020	0.134	0.441	0.761	-0.733	1.001	374
		Q3	-0.067	-0.482	0.461	0.297	-1.388	0.425	374
		Q4	-0.012	-0.082	0.449	0.855	-0.965	0.801	374
	2	Q2	0.031	0.209	0.446	0.640	-0.669	1.087	322
		Q3	-0.024	-0.166	0.462	0.719	-1.075	0.743	322
		Q4	0.031	0.215	0.453	0.636	-0.676	1.105	322
DunedinPACE	1	Q2	-0.042	-0.011	0.015	0.489	-0.041	0.019	410
		Q3	-0.055	-0.015	0.016	0.361	-0.046	0.017	410
		Q4	-0.102	-0.025	0.015	0.098	-0.055	0.005	410
	2	Q2	-0.048	-0.012	0.017	0.463	-0.045	0.021	354
		Q3	-0.036	-0.010	0.017	0.578	-0.044	0.024	354
		Q4	-0.079	-0.020	0.017	0.222	-0.053	0.012	354

Note: St. = Standardized; SE = Standard Error; p = p-value; lowCI = lower 95% confidence interval; upCI = upper 95% confidence interval.

Supplementary Table 13: Linear regression analysis of epigenetic age estimates calculated by all available six epigenetic clocks on quartiles of GPx3 intensity in men and women. Model 1 is unadjusted. Model 2 is adjusted for chronological age, sex, BMI, smoking (packyears), and the first four genetic principal components (PC1 to PC4). The first quartile is used as reference.

Clock	Model		St. β	β	SE	p	lowCI	upCI	n
7-CpG DNAmAA	1	Q2	-0.027	-0.432	0.594	0.467	-1.598	0.734	1066
		Q3	-0.024	-0.390	0.595	0.513	-1.558	0.778	1066
		Q4	-0.040	-0.634	0.593	0.285	-1.797	0.528	1066
	2	Q2	0.012	0.187	0.791	0.813	-1.365	1.740	614
		Q3	0.008	0.127	0.805	0.875	-1.453	1.707	614
		Q4	-0.042	-0.670	0.801	0.403	-2.243	0.903	614
Horvath DNAmAA	1	Q2	-0.033	-0.322	0.462	0.487	-1.228	0.585	689
		Q3	-0.076	-0.772	0.471	0.102	-1.697	0.153	689
		Q4	-0.064	-0.628	0.458	0.171	-1.527	0.272	689
	2	Q2	-0.016	-0.151	0.478	0.753	-1.090	0.788	608
		Q3	-0.048	-0.475	0.489	0.332	-1.436	0.486	608
		Q4	-0.030	-0.284	0.485	0.558	-1.236	0.667	608
Hannum DNAmAA	1	Q2	0.009	0.072	0.377	0.848	-0.668	0.813	689
		Q3	-0.017	-0.140	0.385	0.716	-0.895	0.615	689
		Q4	-0.059	-0.472	0.374	0.208	-1.206	0.263	689
	2	Q2	0.007	0.055	0.390	0.887	-0.712	0.822	608
		Q3	0.002	0.017	0.399	0.967	-0.768	0.801	608
		Q4	-0.021	-0.166	0.396	0.675	-0.943	0.611	608
PhenoAge DNAmAA	1	Q2	-0.034	-0.365	0.498	0.464	-1.343	0.613	689
		Q3	-0.090	-0.989	0.508	0.052	-1.986	0.008	689
		Q4	-0.098	-1.028	0.494	0.038	-1.998	-0.059	689
	2	Q2	-0.031	-0.324	0.521	0.534	-1.346	0.699	608
		Q3	-0.102	-1.087	0.532	0.042	-2.133	-0.042	608
		Q4	-0.061	-0.629	0.527	0.233	-1.665	0.406	608
GrimAge DNAmAA	1	Q2	0.004	0.026	0.332	0.937	-0.625	0.677	689
		Q3	-0.059	-0.430	0.338	0.203	-1.094	0.233	689
		Q4	-0.176	-1.246	0.329	<0.001	-1.892	-0.601	689
	2	Q2	-0.044	-0.313	0.310	0.313	-0.923	0.296	608
		Q3	-0.085	-0.628	0.318	0.048	-1.252	-0.005	608
		Q4	-0.136	-0.975	0.315	0.002	-1.593	-0.358	608
DunedinPACE	1	Q2	-0.006	-0.002	0.011	0.885	-0.023	0.020	764
		Q3	-0.044	-0.011	0.011	0.309	-0.033	0.010	764
		Q4	-0.218	-0.054	0.011	<0.001	-0.076	-0.033	764
	2	Q2	-0.011	-0.003	0.011	0.801	-0.024	0.019	674
		Q3	-0.039	-0.010	0.011	0.369	-0.032	0.012	674
		Q4	-0.146	-0.037	0.011	0.001	-0.059	-0.015	674

Note: St. = Standardized; SE = Standard Error; p = p-value; lowCI = lower 95% confidence interval; upCI = upper 95% confidence interval.

Supplementary Table 14: Linear regression analysis of epigenetic age estimates calculated by all available six epigenetic clocks on quartiles of GPx3 intensity in the subgroup of women. Model 1 is unadjusted. Model 2 is adjusted for chronological age, sex, BMI, smoking (packyears), and the first four genetic principal components (PC1 to PC4). The first quartile is used as reference.

Clock	Model		St. β	β	SE	p	lowCI	upCI	n
7-CpG DNAmAA	1	Q2	-0.115	-1.804	0.832	0.031	-3.439	-0.170	548
		Q3	-0.076	-1.159	0.814	0.155	-2.758	0.440	548
		Q4	-0.043	-0.638	0.793	0.421	-2.195	0.919	548
	2	Q2	-0.052	-0.840	1.124	0.455	-3.051	1.372	324
		Q3	0.012	0.198	1.136	0.862	-2.038	2.433	324
		Q4	-0.028	-0.406	1.058	0.701	-2.487	1.675	324
Horvath DNAmAA	1	Q2	-0.012	-0.120	0.650	0.853	-1.398	1.157	362
		Q3	-0.024	-0.242	0.666	0.717	-1.552	1.068	362
		Q4	0.016	0.144	0.604	0.812	-1.044	1.332	362
	2	Q2	0.007	0.074	0.693	0.915	-1.289	1.438	321
		Q3	0.016	0.165	0.708	0.816	-1.229	1.559	321
		Q4	0.055	0.493	0.652	0.451	-0.791	1.776	321
Hannum DNAmAA	1	Q2	0.019	0.141	0.493	0.774	-0.828	1.111	362
		Q3	0.002	0.016	0.505	0.974	-0.977	1.010	362
		Q4	-0.025	-0.169	0.458	0.713	-1.070	0.733	362
	2	Q2	0.061	0.452	0.521	0.387	-0.574	1.477	321
		Q3	0.058	0.448	0.533	0.401	-0.600	1.496	321
		Q4	0.009	0.058	0.491	0.906	-0.907	1.023	321
PhenoAge DNAmAA	1	Q2	-0.032	-0.348	0.711	0.625	-1.746	1.051	362
		Q3	-0.039	-0.436	0.729	0.550	-1.869	0.998	362
		Q4	-0.069	-0.676	0.661	0.307	-1.976	0.624	362
	2	Q2	-0.048	-0.519	0.751	0.490	-1.998	0.959	321
		Q3	-0.072	-0.801	0.768	0.298	-2.312	0.711	321
		Q4	-0.043	-0.417	0.707	0.555	-1.809	0.974	321
GrimAge DNAmAA	1	Q2	-0.016	-0.106	0.426	0.804	-0.943	0.731	362
		Q3	-0.017	-0.112	0.436	0.797	-0.971	0.746	362
		Q4	-0.131	-0.777	0.396	0.050	-1.555	0.002	362
	2	Q2	-0.066	-0.439	0.437	0.316	-1.300	0.421	321
		Q3	-0.068	-0.469	0.447	0.295	-1.348	0.411	321
		Q4	-0.135	-0.815	0.412	0.049	-1.625	-0.005	321
DunedinPACE	1	Q2	-0.095	-0.022	0.014	0.114	-0.050	0.005	407
		Q3	-0.073	-0.018	0.014	0.221	-0.046	0.011	407
		Q4	-0.308	-0.067	0.013	<0.001	-0.093	-0.041	407
	2	Q2	-0.096	-0.023	0.014	0.113	-0.051	0.005	361
		Q3	-0.089	-0.021	0.014	0.140	-0.050	0.007	361
		Q4	-0.234	-0.051	0.014	<0.001	-0.078	-0.024	361

Note: St. = Standardized; SE = Standard Error; p = p-value; lowCI = lower 95% confidence interval; upCI = upper 95% confidence interval.

Supplementary Table 15: Linear regression analysis of epigenetic age estimates calculated by all available six epigenetic clocks on quartiles of GPx3 intensity in the subgroup of men. Model 1 is unadjusted. Model 2 is adjusted for chronological age, sex, BMI, smoking (packyears), and the first four genetic principal components (PC1 to PC4). The first quartile is used as reference.

Clock	Model		St. β	β	SE	p	lowCI	upCI	n
7-CpG DNAmAA	1	Q2	0.055	0.855	0.826	0.301	-0.767	2.478	518
		Q3	0.037	0.608	0.849	0.474	-1.060	2.277	518
		Q4	-0.020	-0.343	0.874	0.695	-2.059	1.373	518
	2	Q2	0.064	0.992	1.124	0.378	-1.221	3.205	290
		Q3	-0.014	-0.221	1.160	0.849	-2.504	2.063	290
		Q4	-0.068	-1.196	1.248	0.338	-3.652	1.260	290
Horvath DNAmAA	1	Q2	-0.054	-0.510	0.635	0.423	-1.760	0.740	327
		Q3	-0.134	-1.296	0.645	0.045	-2.564	-0.028	327
		Q4	-0.093	-0.999	0.696	0.152	-2.369	0.371	327
	2	Q2	-0.036	-0.324	0.667	0.628	-1.637	0.990	287
		Q3	-0.121	-1.127	0.688	0.103	-2.482	0.227	287
		Q4	-0.124	-1.296	0.741	0.082	-2.756	0.163	287
Hannum DNAmAA	1	Q2	0.001	0.008	0.546	0.988	-1.065	1.081	327
		Q3	-0.038	-0.317	0.554	0.567	-1.406	0.772	327
		Q4	-0.021	-0.190	0.598	0.750	-1.367	0.986	327
	2	Q2	-0.037	-0.299	0.594	0.615	-1.467	0.870	287
		Q3	-0.047	-0.385	0.612	0.530	-1.591	0.820	287
		Q4	-0.040	-0.366	0.659	0.580	-1.664	0.932	287
PhenoAge DNAmAA	1	Q2	-0.037	-0.381	0.694	0.584	-1.746	0.985	327
		Q3	-0.143	-1.514	0.704	0.032	-2.899	-0.128	327
		Q4	-0.094	-1.101	0.761	0.149	-2.598	0.395	327
	2	Q2	-0.010	-0.095	0.729	0.896	-1.531	1.341	287
		Q3	-0.145	-1.489	0.752	0.049	-2.970	-0.007	287
		Q4	-0.076	-0.873	0.810	0.282	-2.469	0.722	287
GrimAge DNAmAA	1	Q2	0.022	0.152	0.457	0.740	-0.747	1.051	327
		Q3	-0.109	-0.763	0.464	0.101	-1.675	0.149	327
		Q4	-0.137	-1.060	0.501	0.035	-2.045	-0.075	327
	2	Q2	-0.035	-0.240	0.447	0.592	-1.121	0.640	287
		Q3	-0.126	-0.873	0.461	0.059	-1.781	0.035	287
		Q4	-0.147	-1.146	0.497	0.022	-2.124	-0.168	287
DunedinPACE	1	Q2	0.080	0.020	0.016	0.209	-0.011	0.051	357
		Q3	-0.015	-0.004	0.016	0.809	-0.036	0.028	357
		Q4	-0.080	-0.022	0.017	0.200	-0.056	0.012	357
	2	Q2	0.064	0.016	0.017	0.345	-0.017	0.049	313
		Q3	0.006	0.001	0.017	0.934	-0.033	0.036	313
		Q4	-0.061	-0.017	0.018	0.354	-0.053	0.019	313

Note: St. = Standardized; SE = Standard Error; p = p-value; lowCI = lower 95% confidence interval; upCI = upper 95% confidence interval.

Figure:

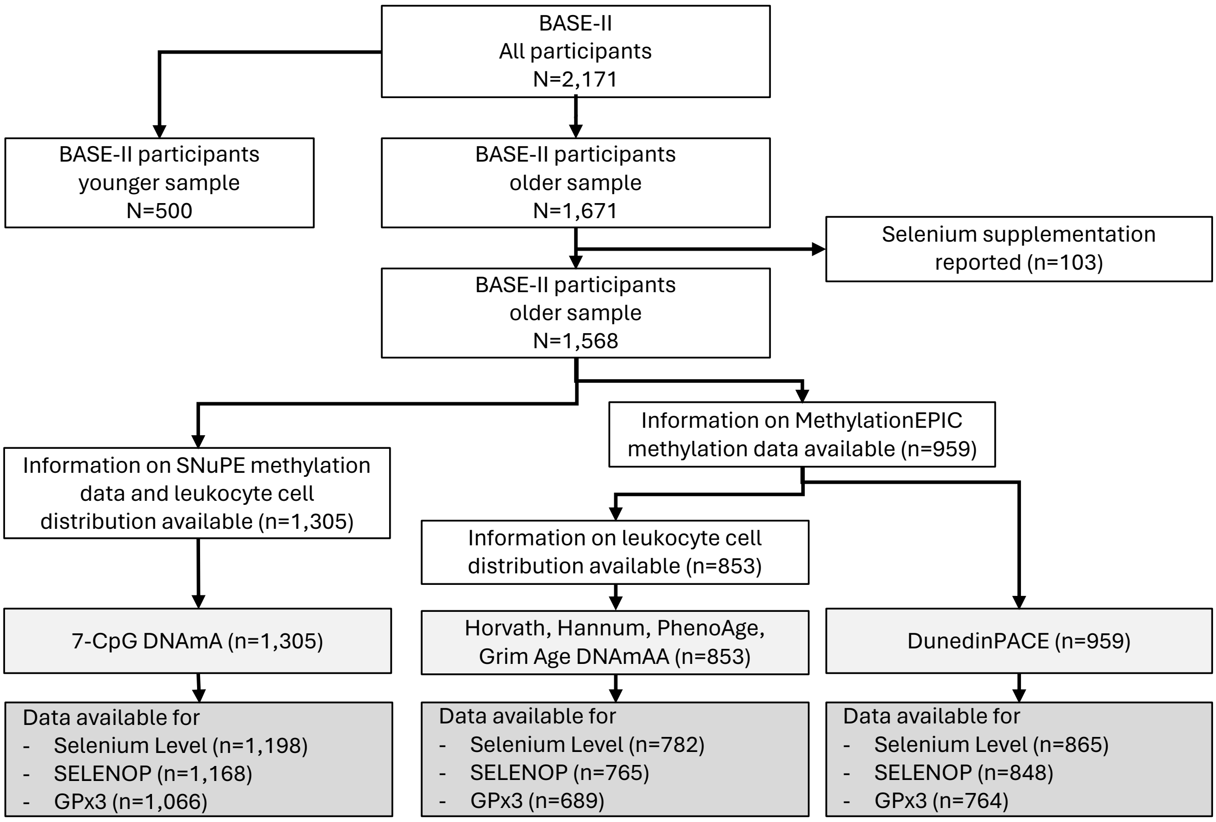

Supplementary Figure 1: Flow-chart of BASE-II participants analyzed in this study.

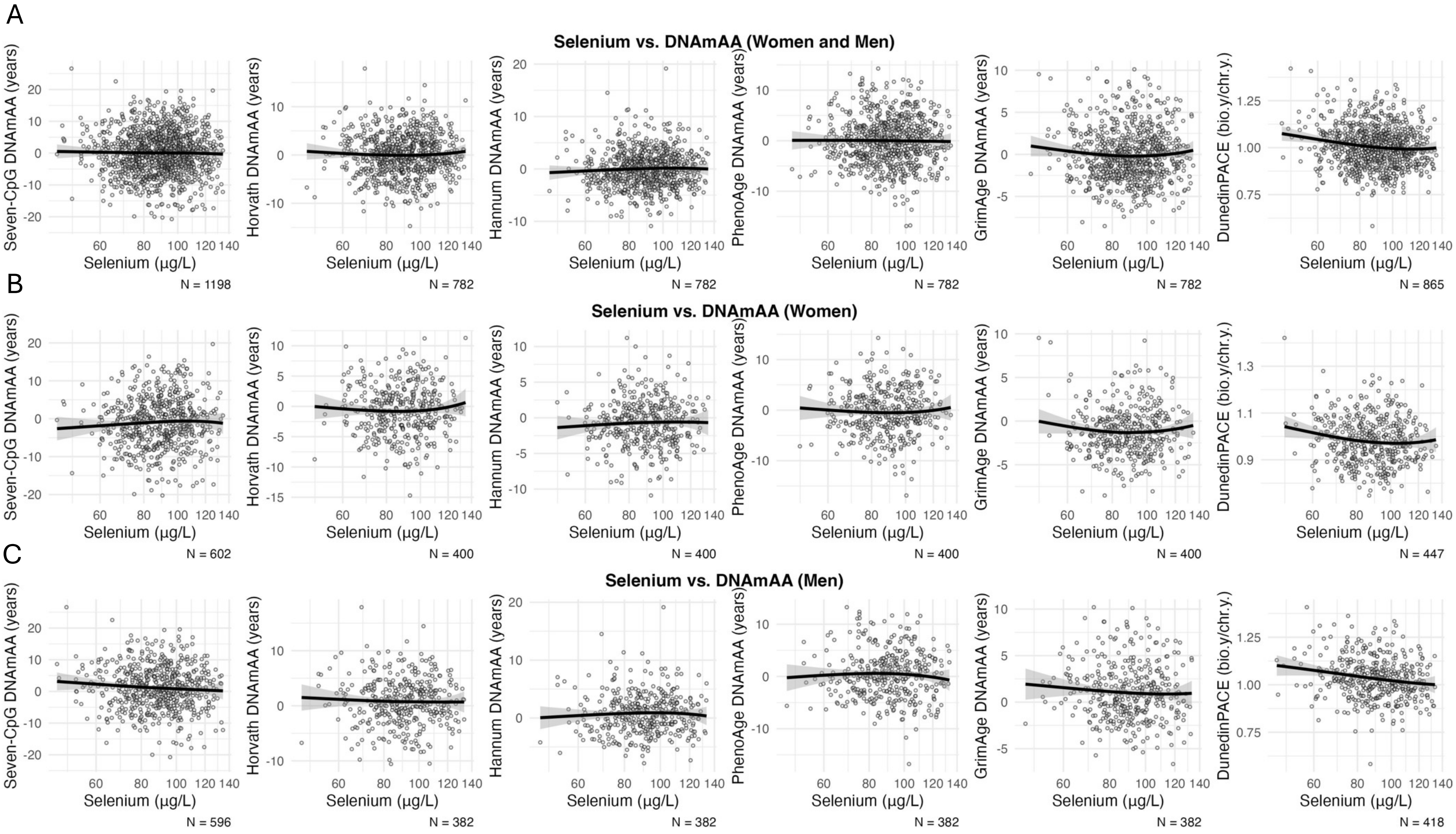

Supplementary Figure 2: Scatterplots of serum selenium levels and biological age estimators calculated from all six available epigenetic clocks in women and men (A) as well as in the all-women (B) and all-me (C) subgroup. The x-axis is log-scaled. All available participants of the older age group are included.

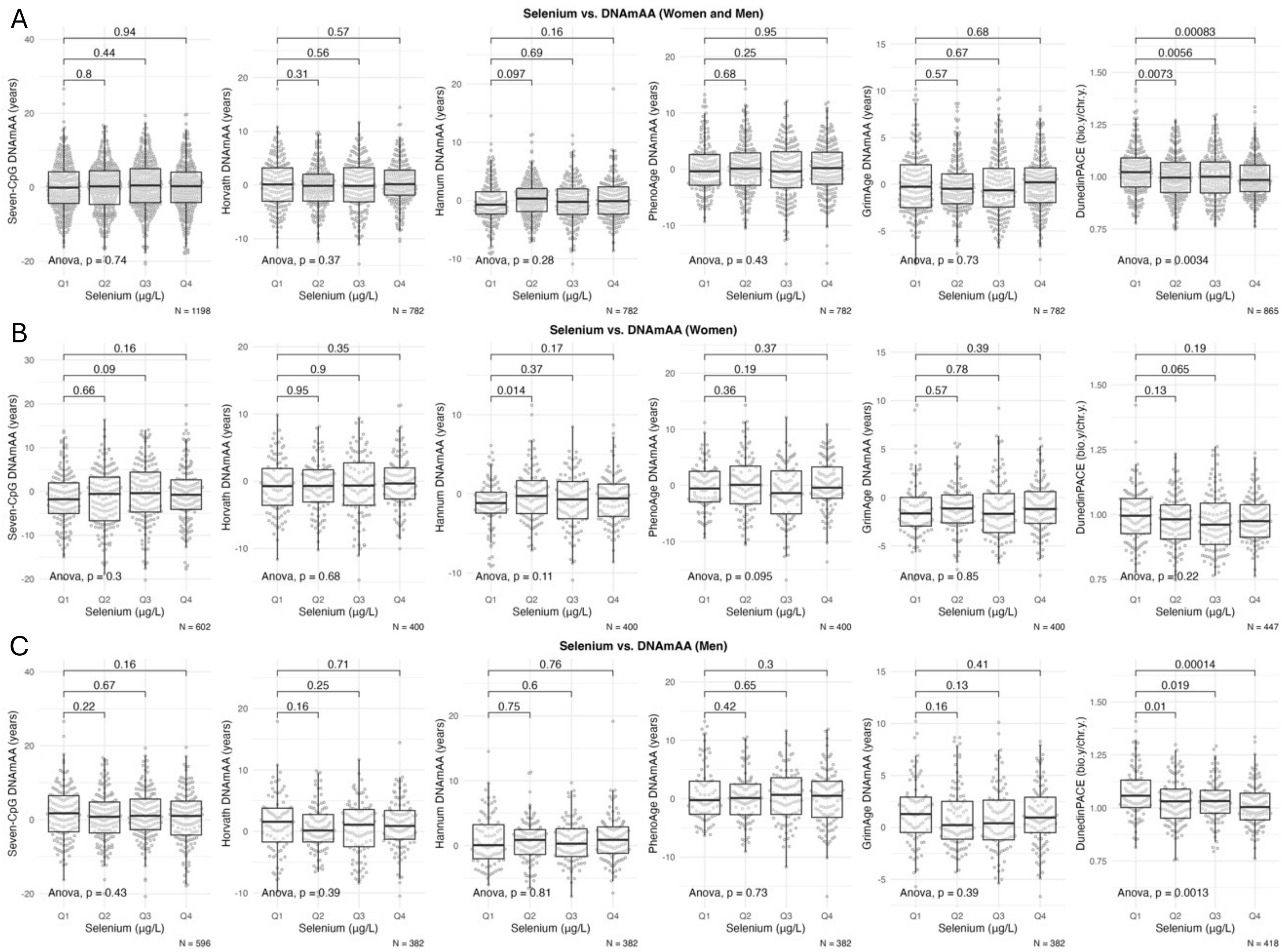

Supplementary Figure 3: Boxplots of biological age estimators calculated by all six available epigenetic clocks stratified serum selenium status (deficient vs. sufficient) in men and women (A), women (B) and men (C). Statistical significance of difference between group means was assessed by t-test.

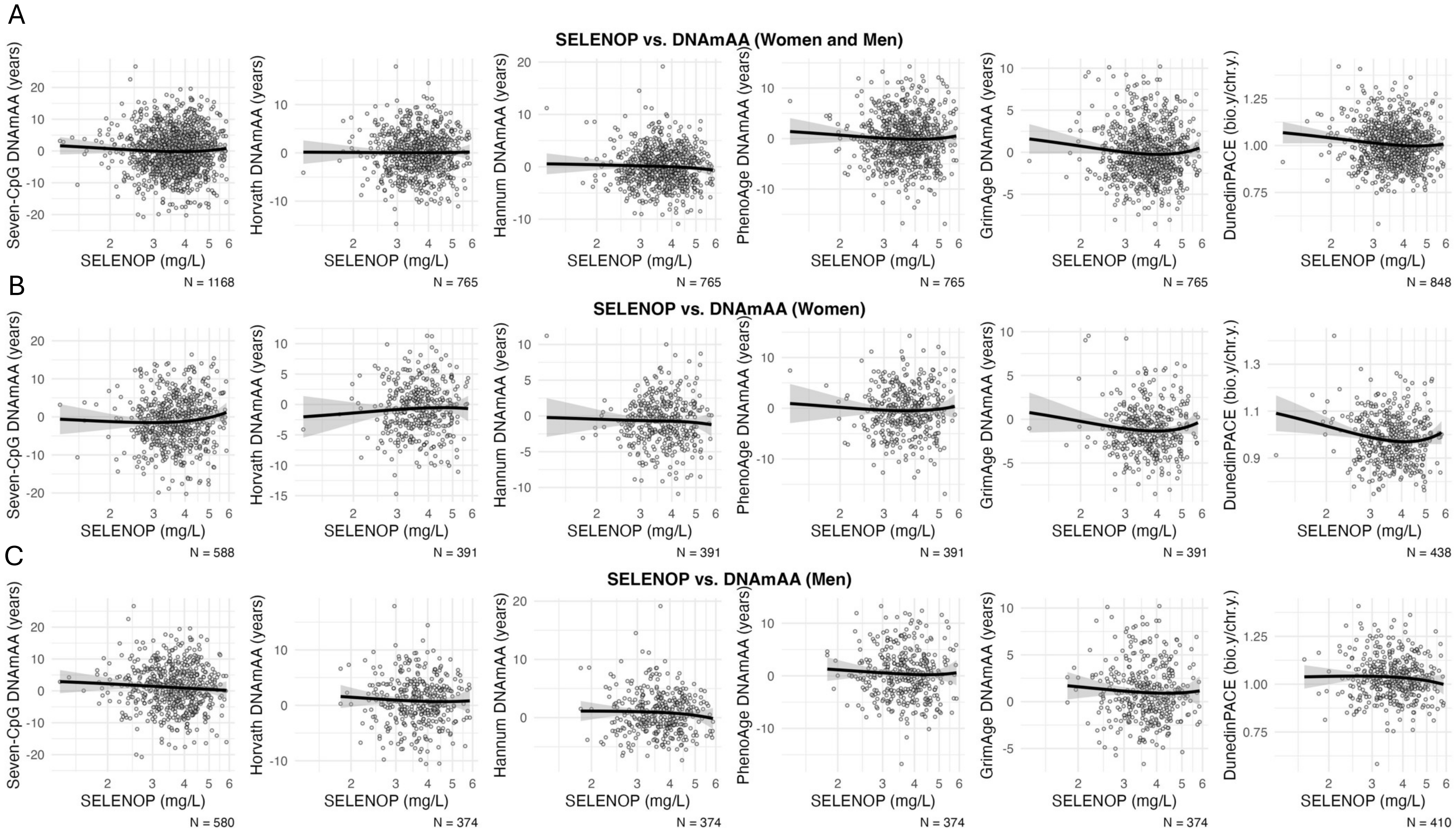

Supplementary Figure 4: Scatterplots of selenoprotein P levels and biologcal age estimators calculated from all six available epigenetic clocks in women and men (A) as well as in the all-women (B) and all-me (C) subgroup. The x-axis is log-scaled. All available participants of the older age group are included.

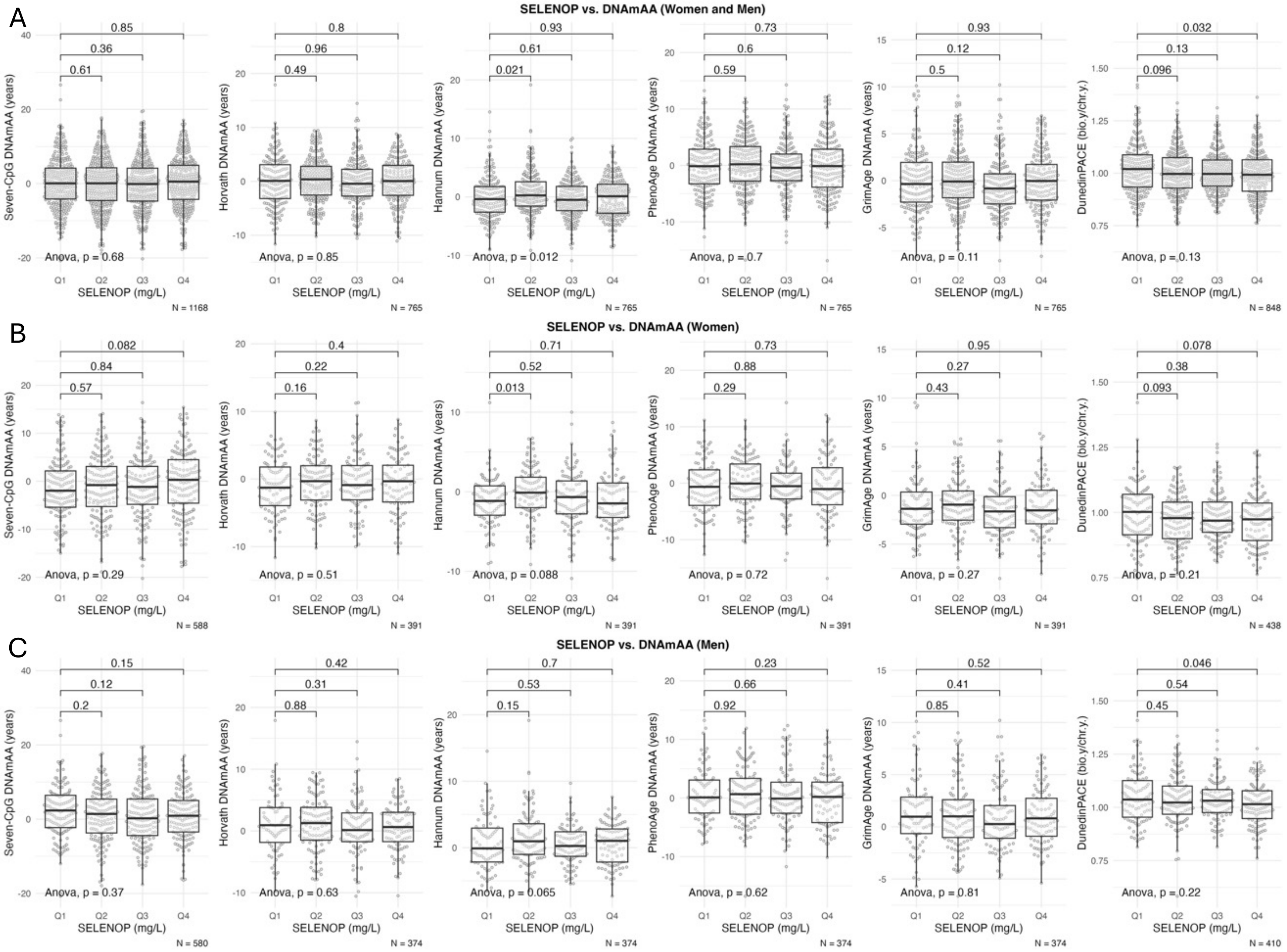

Supplementary Figure 5 Boxplots of biological age estimators calculated by all six available epigenetic clocks stratified by quartiles of selenoprotein P in men and women (A), women (B) and men (C). Statistical significance of difference between group means was assessed by t-test.

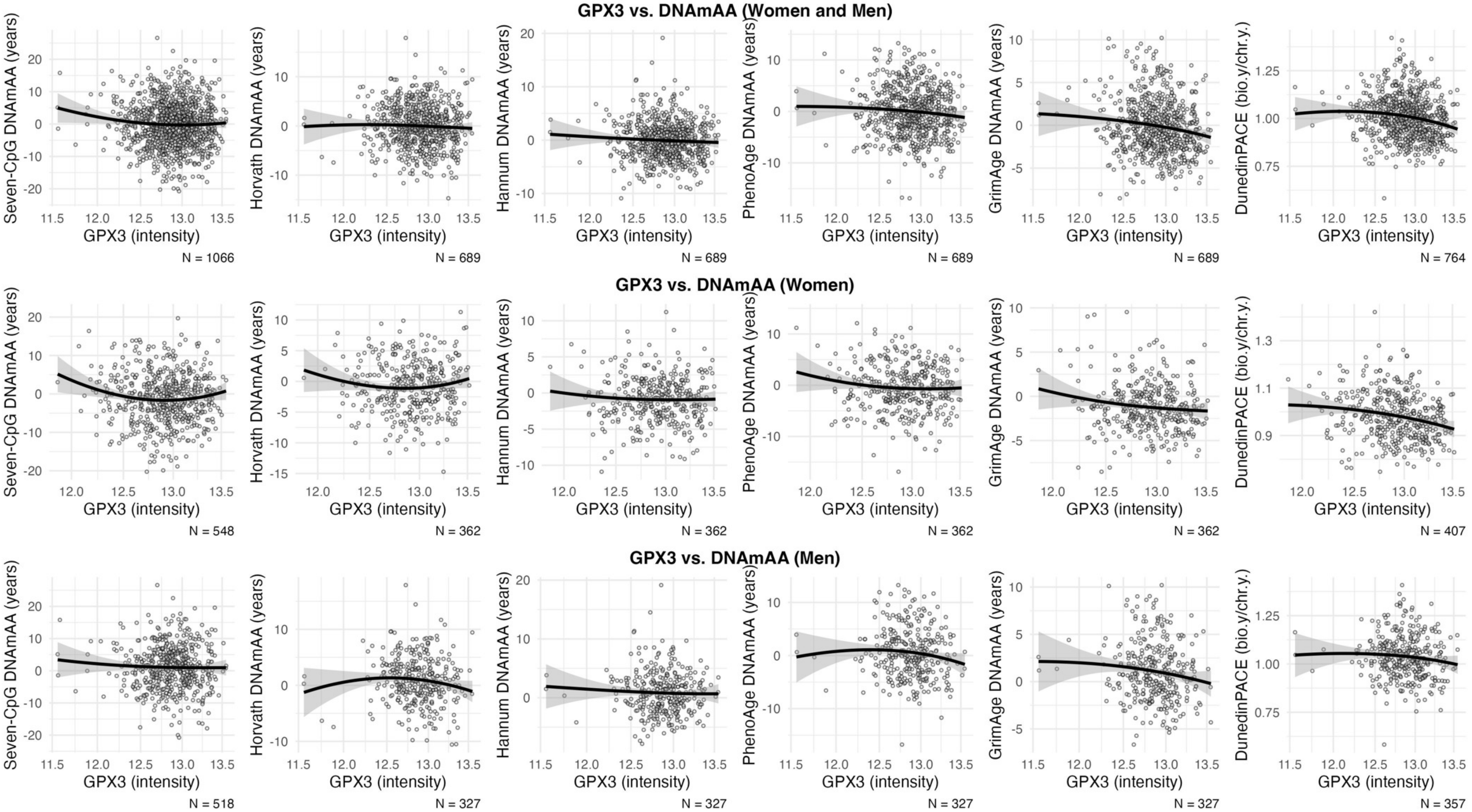

Supplementary Figure 6: Scatterplots of GPx3 intensity and biological age estimators calculated from all six available epigenetic clocks in women and men (A) as well as in the all-women (B) and all-me (C) subgroup. The x-axis is log-scaled. All available participants of the older age group are included.

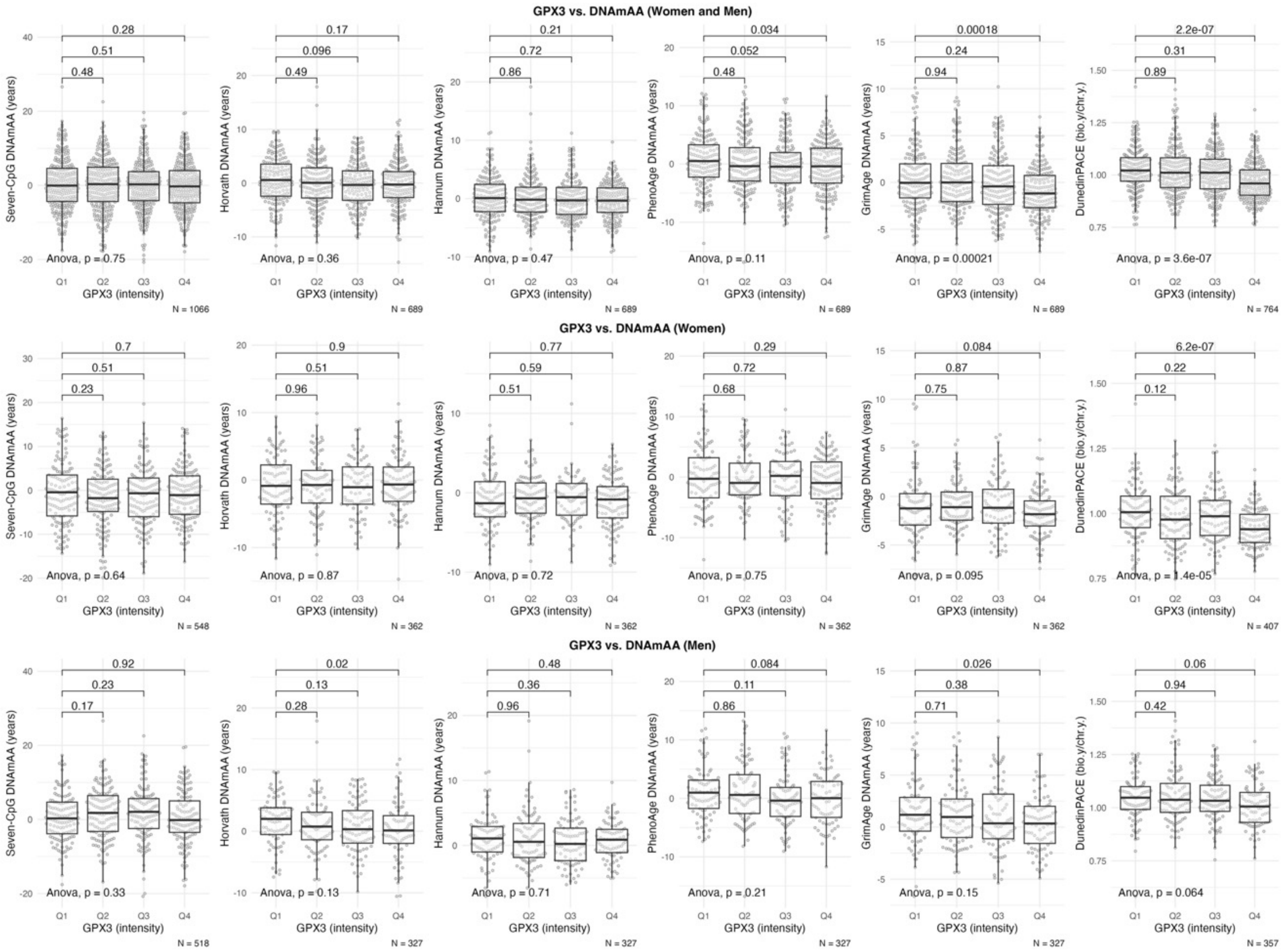

Supplementary Table 7: Boxplots of biological age estimators calculated by all six available epigenetic clocks stratified by quartiles of GPx3 intensities (proteomics) in men and women (A), women (B) and men (C). Statistical significance of difference between group means was assessed by t-test.
